# Regression convolutional neural network models implicate peripheral immune regulatory variants in the predisposition to Alzheimer’s disease

**DOI:** 10.1371/journal.pcbi.1012356

**Published:** 2024-08-26

**Authors:** Easwaran Ramamurthy, Snigdha Agarwal, Noelle Toong, Heather Sestili, Irene M. Kaplow, Ziheng Chen, BaDoi Phan, Andreas R. Pfenning

**Affiliations:** 1 Computational Biology Department, Carnegie Mellon University, Pittsburgh, Pennsylvania, United States of America; 2 Department of Biological Sciences, Carnegie Mellon University, Pittsburgh, Pennsylvania, United States of America; 3 Neuroscience Institute, Carnegie Mellon University, Pittsburgh, Pennsylvania, United States of America; Thomas Jefferson University, UNITED STATES OF AMERICA

## Abstract

Alzheimer’s disease (AD) involves aggregation of amyloid β and tau, neuron loss, cognitive decline, and neuroinflammatory responses. Both resident microglia and peripheral immune cells have been associated with the immune component of AD. However, the relative contribution of resident and peripheral immune cell types to AD predisposition has not been thoroughly explored due to their similarity in gene expression and function. To study the effects of AD-associated variants on *cis*-regulatory elements, we train convolutional neural network (CNN) regression models that link genome sequence to cell type-specific levels of open chromatin, a proxy for regulatory element activity. We then use *in silico* mutagenesis of regulatory sequences to predict the relative impact of candidate variants across these cell types. We develop and apply criteria for evaluating our models and refine our models using massively parallel reporter assay (MPRA) data. Our models identify multiple AD-associated variants with a greater predicted impact in peripheral cells relative to microglia or neurons. Our results support their use as models to study the effects of AD-associated variants and even suggest that peripheral immune cells themselves may mediate a component of AD predisposition. We make our library of CNN models and predictions available as a resource for the community to study immune and neurological disorders.

## Author summary

Human genetic association studies continue to identify a large number of genetic variants associated with common diseases. However, genetic linkage renders it difficult to identify truly causal variants from these associations. The problem is further complicated by the fact that most disease-associated variants lie in non-coding regions of the genome which are involved in regulating gene expression. Gene regulatory elements are highly tissue- and cell-type-specific which makes it important to study disease variant effects in the right tissue or cell type. In this study, we propose a computational framework to identify functional variants that influence disease predisposition by disruption of gene regulatory element activity. We first identify potential cell types that may influence disease predisposition through cell-type-specific gene regulatory element activity. Then, we use machine learning (ML) models that relate human genetic sequence to cell-type-specific gene regulatory activity to characterize potential effects of genetic variants. Finally, using *in silico* mutagenesis, we identify putative functional variants and predict their ability to disrupt regulatory activity in various cell types. Further, we define best practices to train, benchmark, improve and apply these models to interpret the cell-type-specific determinants of disease. In a specific use case, we apply this framework to interpret the cell type-specific contributions of regulatory genetic variants in Alzheimer’s Disease (AD). For AD, we show that we can separate out peripheral immune and brain resident immune microglial signals to understand their specific contributions to AD predisposition. We find that gene regulatory elements active in peripheral immune cells strongly colocalize with AD genetic variants compared to regulatory elements active in neural cell types. In addition, our models predict that many functional AD variants have variant effects that are specific to peripheral immune cells and not brain resident immune cells (microglia). Our study opens up opportunities to experimentally study Alzheimer’s Disease variants in peripheral immune cell models that are often easier to manipulate than many neural cell types and yet not usually considered in functional studies on AD variants.

## Introduction

Alzheimer’s disease (AD) is a progressive age-associated neurological disorder that is characterized by neuron loss and cognitive decline. Within the brains of AD patients, aggregates of amyloid beta, aggregates of tau, and neuroinflammation can often be found well before the clinical phenotypes [1]. Although the pathology of AD is neural, there is mounting evidence that AD pathogenesis is mediated in part by blood-derived cell-types, especially monocytes and macrophages. During the course of normal inflammation, monocytes from the blood infiltrate into damaged tissue and differentiate into macrophages that engulf damaged cells [2]. In the case of inflammation associated with AD, monocytes cross the blood/brain barrier, differentiate into macrophages and strongly impact several molecular features of AD progression [3]. These molecular functions include features that could slow AD progression, such as the clearance of amyloid beta [3,4]. However, there are also well documented neurotoxic effects that could speed up disease progression, including the induction of pathways that increase oxidative stress and secretion of proinflammatory cytokines [5]. The biological model is further complicated by the fact that these functions of peripheral immune cells from the blood are also performed by resident microglia, immune cells derived from a distinct lineage [6]. The relative roles of neurons, microglia and peripheral immune cells in AD predisposition and progression is still largely unknown.

Genome-wide association studies for AD have the potential to link AD predisposition to the different components of its pathology. Thus far, these studies have identified 30–40 known risk loci for Alzheimer’s disease, with hundreds more implicated at lower confidence [7–11]. Despite this wealth of information, identifying candidate functional AD-associated SNPs (FAD-SNPs) remains a challenge. First, linkage disequilibrium (LD) can lead to many (up to hundreds) genetic variants at a particular locus being highly correlated. Usually, the most strongly associated variants (or sentinel variants) at disease associated loci are strong candidates for being causal variants, but it is often impossible to definitively identify which variant (s) in a region are responsible for the association signal [12]. The second challenge for interpreting GWAS for AD and other studies is functional interpretation. For example, in a recent AD GWAS [8], only 2% of variants in genome-wide significant loci (p<5x10^-8^) are within known exon annotations. Instead, a large proportion of disease-associated variants lay in putative promoter and enhancer gene regulatory elements. In fact, 69% lay in enhancer states, 26% lay in regions containing promoter-associated histone marks, and 46% lay in DNase I hypersensitive sites from a large set of cell types and tissues in the HaploReg database [13]. Therefore, it is likely that FAD-SNPs affect the transcription of nearby genes through the alteration of regulatory element activity. Usually, this occurs by disruption of DNA sequence patterns (or motifs), which enable transcription factor (TF) binding to DNA [14]. Sites of TF binding in the genome and their regulatory activities can be measured using technologies such as chromatin immunoprecipitation and sequencing (ChIP-seq) for transcription factors and histone modifications, and chromatin accessibility assays such as DNase I or transposase accessible site sequencing (DNase-seq/ATAC-seq) [15]. Notably, these distal regulatory elements are highly cell type- and tissue-specific, making them a useful tool in linking the genetic loci to function [16].

Comparisons of AD GWAS to cell type- and tissue-specific epigenomic studies have highlighted the importance of the immune system in AD predisposition. Multiple studies on a variety of GWAS and regulatory datasets have found that enhancers in certain peripheral immune cells, including monocytes, are more enriched for AD-associated genetic variants than brain tissue [17,18]. In parallel, enrichments that are just as strong have been discovered in isolated and cultured microglia [18–21]. For specific loci, these annotations have helped to identify the most likely function of SNPs within the locus [18,21,22]. However, the developmental and functional similarities between peripheral monocytes and macrophages and resident microglia have made systematically dissecting the relative contributions of these cell types using standard methods difficult [23].

Several experimental methods have attempted to assign regulatory function to genetic variants. Expression quantitative trait loci (eQTL) studies have attempted to address this by identifying genetic variants associated with transcription levels of genes, but these suffer from the same LD confounds that GWAS does, so, in many cases, one cannot determine which of multiple tightly linked regulatory elements affect gene expression. In the case of AD, eQTLs have been used to identify the function of candidate loci [24,25] and have further implicated peripheral immune cell subtypes [26], but the lack of available data in microglia makes a direct comparison difficult. Profiling of regulatory elements and careful assessment of variant effects on measured regulatory elements also could help identify functional and potentially causal variants. However, most profiling of regulatory elements has been conducted in a small number of non-diseased individuals, and profiling regulatory elements in large numbers of individuals with a disease of interest is usually infeasible due to high cost and challenges in collecting post-mortem tissue. This infeasibility leads to low variety of cell types and low statistical power in chromatin accessibility quantitative trait loci (caQTL) [27] and histone acetylation QTL (haQTL) [28] studies.

Machine learning (ML)-based approaches are emerging as a feasible technique to interpret candidate regulatory disease-associated loci without the need for generating large datasets in specific cells. Building upon the availability of regulatory genomics datasets in non-diseased individuals, recent studies have developed machine learning models including convolutional neural networks (CNNs) [29–31] and gapped k-mer support vector machines (SVMs) [32–34] that can accurately predict regulatory potential of an input genome sequence in a given a tissue, cell type, or context. Compared to motif scanning and position weight matrix (PWM) scanning based approaches, these models, particularly CNNs, can automatically learn motifs as well as non-linear interactions between different TF binding sites without requiring manual design of input features. These models have been shown to be more accurate than linear models whose features are known motifs. Typically, these more sophisticated models rely on the inherent genetic variation across a single human reference genome to learn the code that relates sequence to regulatory activity in a tissue, cell type, or context of interest. By learning this regulatory code, these models are capable of predicting the effect of genetic variants on regulatory activity *in silico*. Further, by direct prediction of variant effect on the overlapping regulatory element, these models are capable of interpreting signals from GWAS in regulatory loci and identifying the cell types, tissues, and contexts in which GWAS variants have an effect [20].

**Fig 1:**
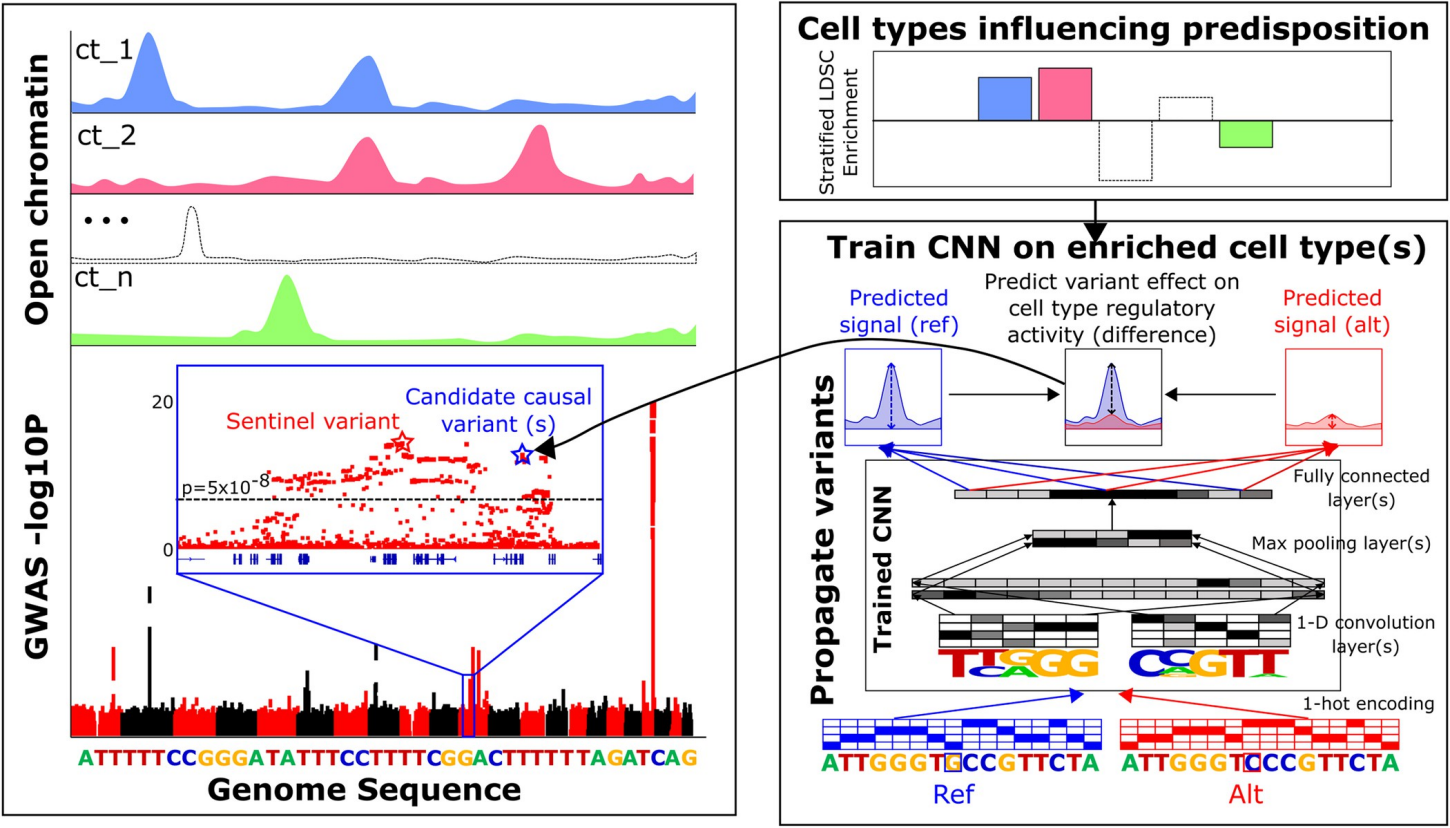
Overview of framework In step 1, we integrate GWAS data with open chromatin profiles and use partitioned heritability analysis to identify cell types that may influence predisposition to disease through variant effects on gene regulatory elements. In step 2, we model open chromatin signal values as a function of genomic sequence using convolutional neural network (CNN) models trained on open chromatin profiles from disease relevant cell types. In step 3, we use the trained CNNs to score disease associated variants for variant effects.

We build upon these studies to construct a framework for identifying putative functional regulatory variants for a disease and the cell types in which they are likely to affect the disease **(Fig 1)**. In the first step, we use stratified LD score regression (S-LDSC) [35–37] on open chromatin measurements from large sets of epigenomic data to identify disease relevant cell types and tissues in which transcriptional regulation is likely to contribute to disease predisposition. Then, we train CNN models on regulatory annotations in high-scoring cell types that enable the prediction of the effects of genetic variants on regulatory activity. We provide guidance on how to train these models by performing a rigorous evaluation of the performance of CNN models trained on open chromatin data to predict both regulatory activity of sequences and regulatory effects of genetic variants in a large MPRA study [38]. We do this for both classification models and regression models and for models trained on both DNase-seq and MPRA data. We find that we can improve our models by using transfer learning, in which we pre-train models on open chromatin datasets and then fine tune these models on MPRA sequences. Using our model predictions, we identify which of multiple variants in LD are likely to be functional as well as the regulatory elements and cell types in which these putatively functional variants influence transcriptional regulation.

We apply our framework to solve the dual problem of predicting candidate FAD-SNP effects and identifying the cell types through which they are most likely to act. Although previous studies have revealed that open chromatin regions in microglia are enriched for common variants associated with AD [19,21], in a direct comparison between the two cell types using our S-LDSC analysis, we find that peripheral blood CD14+- monocyte regulatory elements are strongly enriched for colocalization with AD-associated genetic variants compared to brain resident microglial regulatory elements. We interpret the function of AD-associated genetic variants in regulatory loci that are specifically active in the peripheral blood cell types and brain resident microglia and find candidate variants that our model predicts to influence AD predisposition through disruption of regulatory activity specifically in peripheral immune cell types.

## Results

### CNN regression models and CNN classifiers trained on trained on LCL open chromatin data can accurately predict open chromatin signal from sequence

**Fig 2:**
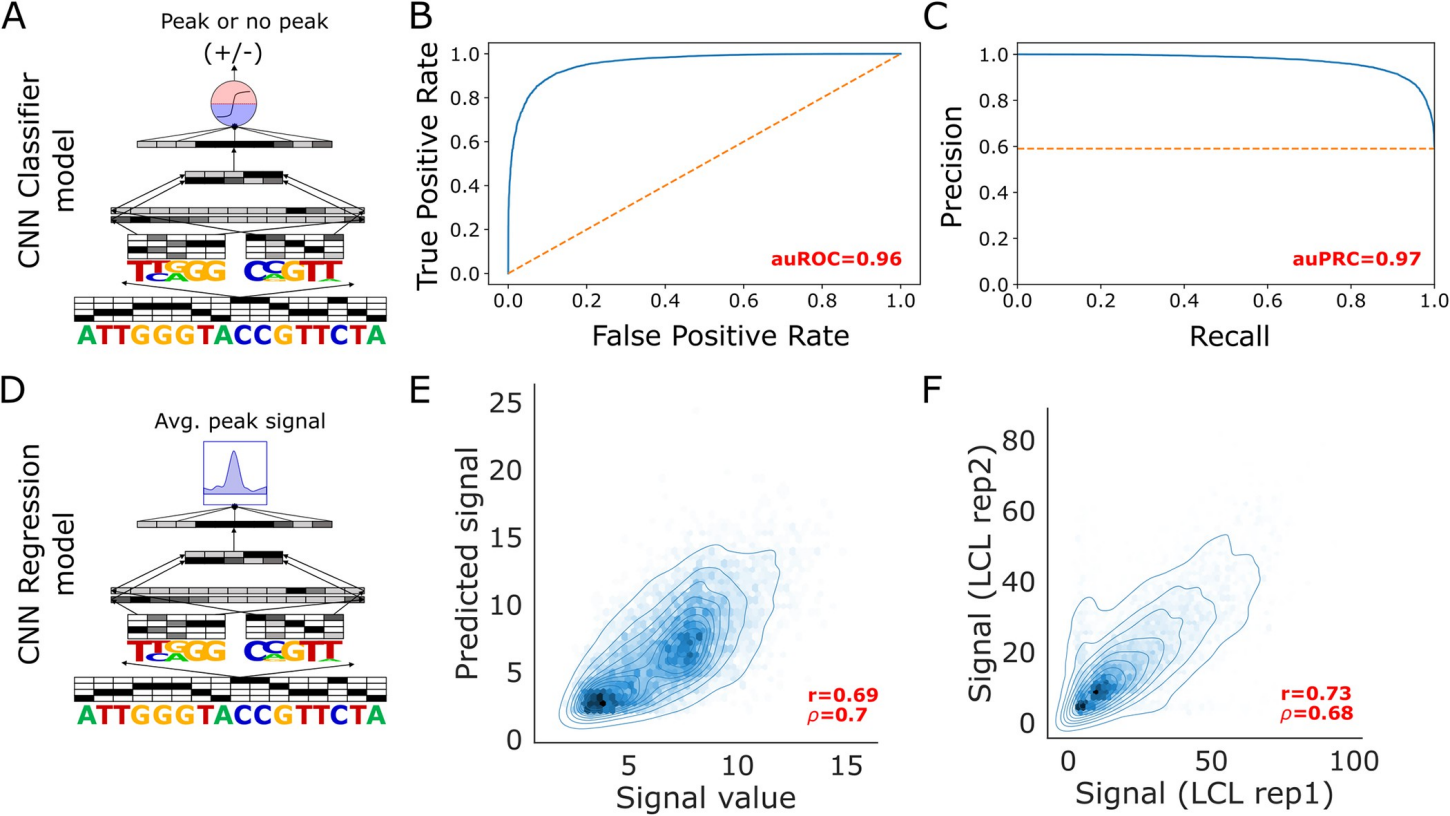
Generalization performance of CNN classifiers and regression models trained on LCL DNase-seq data A. cartoon diagram of a CNN classifier that we trained. The models takes in a one-hot encoded genomic sequences, passes it through the convolutional network that contains alternating convolutional and max pooling layers and outputs a sigmoid transformed value representing the probability that a sequence underlies an OCR or not. **B.** auROC on test set sequences derived from held out chromosomes (chromosomes 8 and 9) of a classifier trained on LCL DNase-seq OCR peaks and equal numbers of GC matched negative controls **C.** auPRC of CNN classifier on held out test set sequences **D.** cartoon diagram of a CNN regression model that we trained. The model structure is similar but instead of a sigmoid probability value, it is trained to output the quantitative signal from the open chromatin assay (DNase-seq/ATAC- seq) **E.** Scatter plot of CNN regression model predicted signal vs the actual signal value from the peak calling algorithm for chromosome 8 and 9 test set peaks that were held out during training and hyperparameter tuning. Pearson and Spearman correlation between predicted and true signal is included in red text **F.** Scatter plot of true signal values in individual DNase-seq replicates for the same set of peaks presented in E.

To investigate the types of models that work well for our framework, we trained CNN classification and regression models on open chromatin regions (OCRs) identified from 5 DNase-seq datasets of GM12878 lymphoblastoid cell lines (LCLs) in the ENCODE database [39,40]. We chose this cell line because it has publicly available high-throughput MPRA data that includes sequences with different alleles of genetic variants that can be used for variant effect evaluation [38]. We trained the classifiers on positive example sequences underlying OCRs and an almost equal number of negative example sequences derived from closed regions of the genome that were matched for G+C nucleotide content with the positive examples (**Fig 2A**). Our CNNs are similar to previously described CNN classifiers [29–31] and gapped k-mer (gkm) kernel SVM [32,33,41] methods in the literature that model binary labels representing open or closed chromatin. Our CNN classifier was able to accurately generalize to test set OCR and negative examples on chromosomes (chromosomes 8 and 9) not used for training or hyper-parameter tuning (area under receiver operating characteristic auROC = 0.96, area under precision-recall curve auPRC = 0.97) **(Fig 2B and 2C).** Such high performance levels are not unexpected since CNNs have been shown to be extremely accurate at related tasks such as TF binding prediction. Additionally, the auPRC for random guessing is ~0.5 since the classes are approximately balanced i.e. the number of positives is roughly equivalent to the number of negative examples.

In addition, we trained CNN regressions that model quantitative OCR signal instead of a binary label **(Fig 2D)**. Our motivation for doing this is that most common variants are unlikely to fully deplete regulatory activity **(S1 Fig)** and rather might have smaller effects on regulatory activity [42]. Our CNN regression model was also able to generalize to held out examples on chromosome 8 and 9 LCL OCRs with a Pearson’s correlation (r) of 0.69 and Spearman’s correlation (ρ) of 0.7 between the true OCR signal and model predicted OCR signal **(Fig 2E)**. Notably, signal values for the same set of OCRs displayed a moderately higher correlation (r = 0.73, ρ = 0.68) between the two replicates that were used to define the final peak set that went into training **(Fig 2F)**. Given such noise in the experimental system, this is approaching the best performance a model can achieve on this problem. These results suggest that using regression models for our framework is feasible.

### LCL DNase trained CNN models can accurately predict measured regulatory activity in MPRA conducted in LCLs

**Fig 3:**
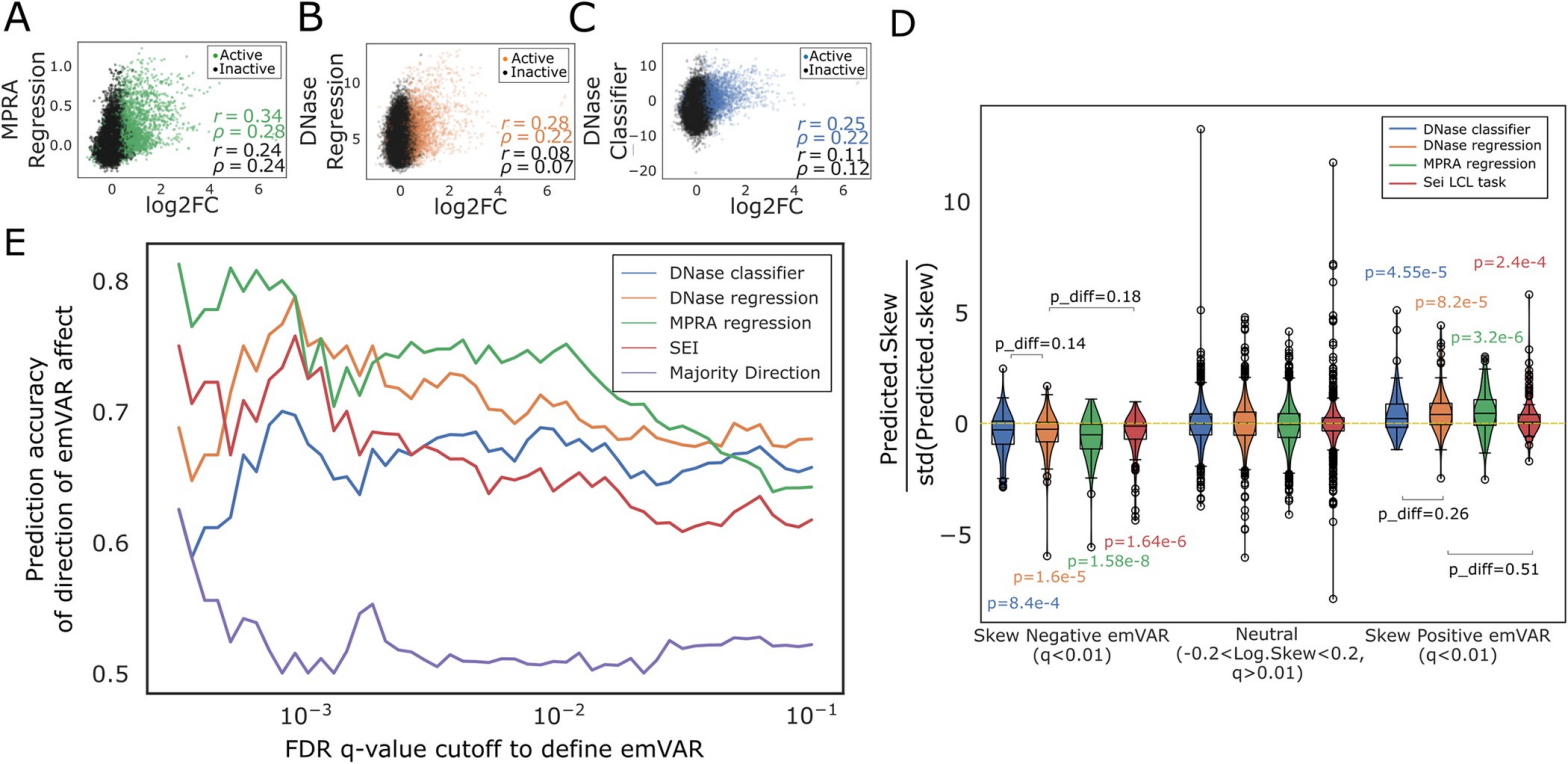
Performance of LCL DNase-trained CNN classifiers, LCL DNase-trained regression models, and MPRA-trained regression models on the task of predicting regulatory activity and variant effects in the Tewhey *et al*. [38] MPRA A. Scatter plot between true and predicted MPRA regulatory activity showing generalization performance of the MPRA-trained model on test sequences held out during training and hyper-parameter tuning of the model. Pearson and Spearman correlation values between true MPRA activity and predicted MPRA activity are indicated in the bottom right for active and inactive sequences in the MPRA study. B. Scatter plot between true MPRA signal and predicted signal from the DNase-trained CNN regression model on the MPRA model test set. Points are colored by whether the sequences were deemed active or inactive in the MPRA and corresponding correlations on these subsets are also reported in the bottom right. C. Similar to B but, instead of the CNN regression, the plot displays performance and predictions from the DNase-trained CNN classifier. The predictions on the y-axis are logit (inverse sigmoid) transformed. D. Violin plot displaying performance of the MPRA-trained model, the DNase-trained classifiers, and the DNase-trained regression on predicting allelic skew (or variant effect) in the MPRA study. Shown are the allelic skew values for three different subsets of variants derived from the MPRA test dataset. These subsets correspond to 1) variants that are confidently (FDR q<0.01) called to have higher MPRA activity for the alternate allele compared to the reference allele (”skew negative emVars”); 2) variants that do not display confident allelic skew in the study (|log(skew)|<0.2 and FDR q>0.01); 3) variants that are confidently called to have lower MPRA activity for the alternate allele compared to the reference allele (FDR q<0.01) (”skew positive emVars”). The p-values from one-sample t-tests comparing distribution shifts from neutrality (0 value) are indicated above or below the violins. The p-values from two sample t-tests (represented pdiff) comparing predictions from the DNase-trained classifier and the DNase-trained regression are also displayed. E. Accuracy of the prediction of the direction of variant effect of the DNase-seq-trained regression, the DNase-seq-trained classifier, the MPRA-trained regression and the best-performing LCL DNase task from the Sei multitask model on variants in the MPRA test dataset. Accuracy is reported at different FDR q-values to call emVars. Accuracy for a naïve baseline classifier that always predicts the direction of effect for a variant to be the direction of effect for majority of variants is also included as a baseline.

Since not all OCRs function as enhancers or promoters [43], we evaluated our model’s performance at predicting direct measurements of enhancer regulatory activity using previously published MPRA data from LCLs in a zero-shot manner [38]. First, we evaluated how well our models were able to predict the regulatory activity (as measured by the log2(RNA/plasmid DNA)) of each sequence included in the MPRA. To conduct an unbiased evaluation, we next trained a separate single-task CNN directly on the MPRA data. It models the MPRA measured regulatory activity (log2(RNA/plasmid DNA)) as a function of the 1000bp extended sequences derived from the MPRA. This differentiates our work from previous studies that either train only on MPRA data [44] or do not compare models trained on assays like DNase-seq against an MPRA-trained model [45]. Our MPRA-trained model was able to achieve a moderate correlation (r = 0.34, ρ = 0.28) between predicted MPRA activity and measured MPRA activity for held out test set sequences that were called active in the MPRA and not used for training or hyperparameter tuning **(Fig 3A)**. Not surprisingly, the MPRA-trained model predictions displayed a lower correlation for the subset of MPRA sequences that were called inactive in the Tewhey *et al*. [38] study (r = 0.24, ρ = 0.24). Interestingly, on the active test set sequences, our DNase trained regression model achieved slightly lower but statistically significant correlations between model predictions and MPRA measured signal (r = 0.28, ρ = 0.22) **(Fig 3B)**. The performance of the DNase-trained CNN classifier was slightly lower than the DNase-trained CNN regression model (r = 0.25, ρ = 0.22) **(Fig 3C**). Notably, MPRA data is very noisy, and enhancer activity is measured outside of the enhancer’s genomic context which can lead to discrepancies between the MPRA measured activity and the true activity of the enhancer in its genomic context. In addition, not all DNase-seq peaks function as enhancers. Therefore, these correlations suggest that the DNase-seq-trained models have some ability to learn relevant signals and generalize to MPRA data.

### CNN models trained on DNase-seq data can predict direction of variant effects in MPRA accurately

Since our final goal is to identify genetic variants that affect enhancer activity, we evaluated our models on their ability to predict the direction of allelic skew for expression modulating genetic variants (emVars). Notably, this is done in a zero-shot manner for DNase-seq trained classifiers and regression models since they are only trained on the wild type reference genome and have not seen any variants during training. We found that the DNase-seq trained classifier and regression achieved performance on par with the MPRA trained model **(Fig 3D).** At most emVar q-value cutoffs, the DNase-trained regression outperformed the DNase trained classifier at predicting the direction of effect of emVars and, at certain cutoffs, it achieved performance on par with the MPRA trained model **(Fig 3E)**. In addition, all three models performed better than a theoretical naive classifier that predicts the direction of effect to be the majority class. Further, we compared our models to the best-performing LCL chromatin accessibility task (highest auPRC) from the Sei multitask model [46], which is an updated version of the DeepSea [29] classification model. We found that the performance of our DNase regression model was consistently above the Sei model across the range of q-value cutoffs for defining emVars **(Fig 3E)**. Notably, the Sei LCL model performed better than our DNase classifier at lower q-value cutoffs but worse at higher q-value cutoffs.

### Stratified LD score regression analysis identifies brain resident microglia and peripheral immune cell types relevant for study of regulatory variation associated with Alzheimer’s Disease

**Fig 4:**
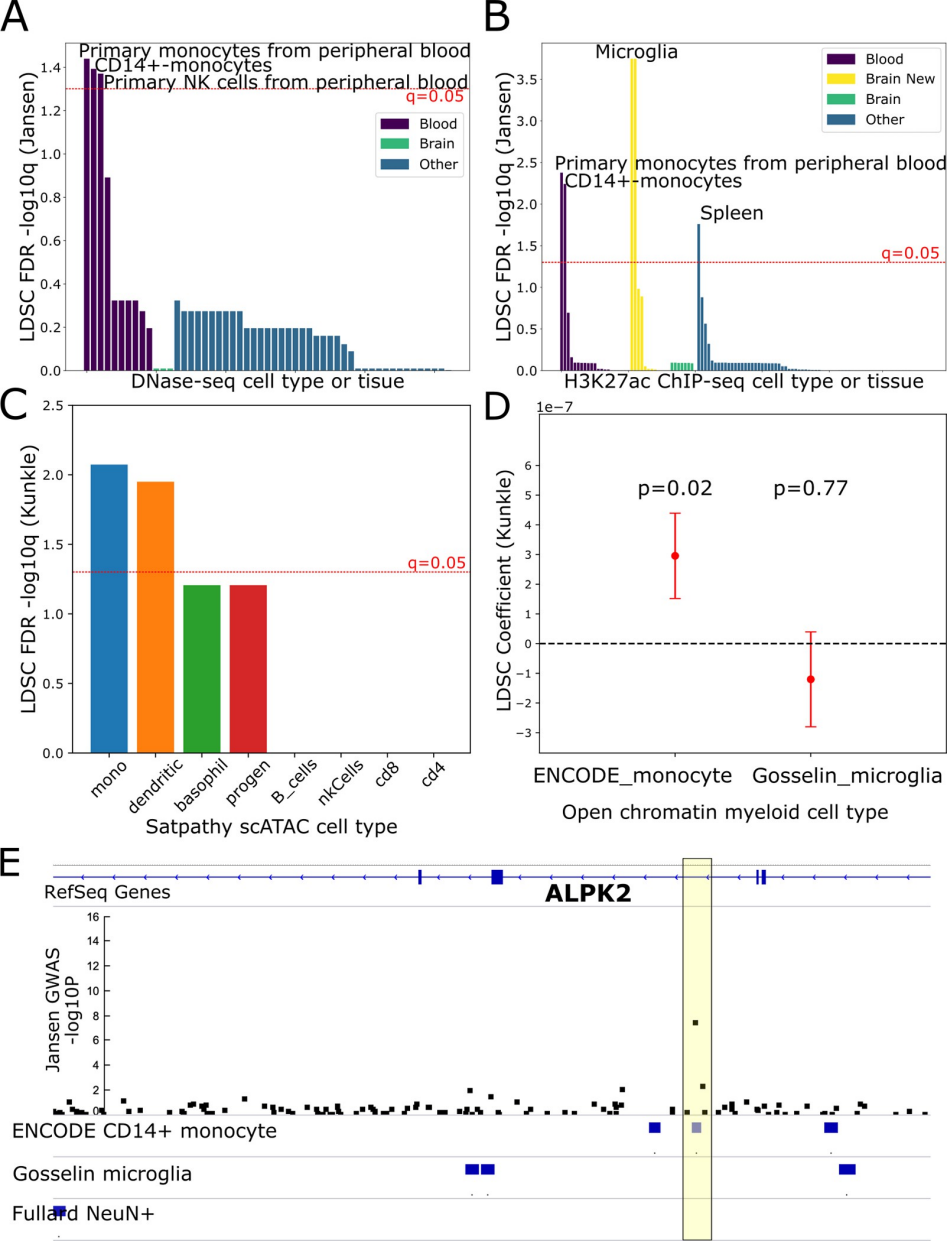
Systematic stratified LD score regression analysis on histone acetylation and open chromatin profiles reveals a stronger AD GWAS enrichment for peripheral immune cell types compared to microglia. A. Bar chart depicting FDR q-values from an S-LDSC analysis on the Jansen *et al*. [9] AD-by-proxy GWAS and included DNase-seq peaks from 53 cell types/tissues in the Roadmap Epigenomics dataset. Red line indicates q = 0.05, the significance cutoff based on FDR. Tissues are categorized and colored by whether they are derived from brain, blood, or other tissues. B. Bar chart depicting FDR q-values from an S-LDSC analysis on the Jansen *et al*. [9] GWAS and H3K27ac ChIP-seq peaks from 98 cell types/tissues in Roadmap Epigenomics dataset as well as 12 profiles (”Brain New”) from the cell type-specific brain H3K27ac dataset from Ramamurthy, Welch *et al*. [19] Tissues are categorized and colored by whether they are derived from brain, the new brain dataset, blood, or other tissues C. Bar chart depicting FDR q-values from S-LDSC analysis on the Kunkle *et al*. GWAS [8] and 8 immune cell type peak sets derived from the scATAC-seq dataset in Satpathy *et al*. [49] mono-Monocyte, dendritic—Dendritic cells, progen—progenitors, basophil—Basophils, nkCells—Natural Killer cells, B_cells—B cells, cd8—CD8+ T cells, cd4—CD4+ T cells D. Dot and whisker plot depicting the LDSC heritability enrichment coefficient from S-LDSC analysis on the Kunkle *et al*. [8] AD GWAS and open chromatin peak sets derived only from the ENCODE monocyte DNase-seq dataset and the Gosselin *et al*. [51] microglia ATAC-seq dataset. P-values for enrichment of each cell type relative to the other are indicated above the dot and whiskers. Whisker ends represent standard errors computed by the S-LDSC software. E. Genome browser view of the locus containing the ALPK2 gene which contains a GWAS association with AD by-proxy in the Jansen *et al*. [9] GWAS. Shown are tracks for RefSeq genes, the p-value (-log10 transformed) from the Jansen *et al*. [9] GWAS, and open chromatin peak annotations for CD14+ monocytes (ENCODE), microglia (Gosselin *et al*. [51]), and putamen neurons (Fullard *et al*. [56]). The yellow box indicates the location of the sentinel AD-associated variant (rs76726049) at this locus. As visualized, this variant overlaps a monocyte peak but not a microglia peak, suggesting a potential peripheral immune-specific function and not a brain related function for this variant. NOTE: Since this is exploratory analysis to identify potentially relevant cell types, in some cases, we show results from the Jansen *et al*. [9] study and in some cases, we show results from the Kunkle *et al*. [8] study. Overall, if we find a positive hit in one S- LDSC analysis, we include it in future model training since it could be relevant to disease. The remaining S-LDSC analysis plots are provided in S2 and S3 Figs.

The first step of our framework involves using S-LDSC to identify cell types that are likely to be associated with disease. We apply this approach to compare the contribution of different neural and immune cell type subtypes to AD predisposition. To do this, we first systematically conducted S-LDSC analysis to test whether heritability for AD was enriched in open chromatin and H3K27ac ChIP-seq profiles in specific cell types/tissues out of a large set of cell types/tissues. We used GWAS summary statistics from two different AD studies: Kunkle *et al*. [8] and Jansen *et al*. [9]. From DNase-seq profiles of 53 different cell types/tissues in the Epigenome Roadmap database [16], we detected statistically significant colocalization between AD-associated variants and open chromatin in peripheral immune and blood cell types, particularly myeloid cell types. This included primary CD14+ monocytes, primary monocytes from peripheral blood, and primary natural killer (NK) cells from peripheral blood **(Fig 4A)**. Notably, microglia are not included in the Epigenome Roadmap DNase-seq datasets at all and, hence, we added microglial datasets to subsequent S-LDSC analyses. First, we conducted S-LDSC analysis on H3K27ac ChIP-seq peaks from all 98 different tissues/cell types downloaded from the Epigenome Roadmap [16]. In addition to these 98 peak sets, we included 12 additional peak sets derived from microglia, oligodendrocyte enriched glia (OEG), and neurons from the hippocampal and cortical H3K27ac ChIP-seq dataset of non-diseased human controls that we have previously analyzed [19]. In this analysis, we again found that multiple peripheral blood cell types were enriched for colocalization with AD risk **(Fig 4B)**. In addition, the microglial H3K27ac peaks displayed a significant colocalization with AD risk, in line with previous work [19,21,47]. Further, the spleen was enriched, which is likely due to the high proportion of monocytes in that tissue [48]. Notably, immune cell type enrichments on the broader H3K27ac peaks are consistent with the enrichment results on the narrower DNase-seq OCRs.

### Monocytes and dendritic cell open chromatin regions are enriched for AD-associated regulatory variants relative to other immune cell types

To compare cell type enrichments within different immune cell types, we reprocessed the single-cell ATAC-seq dataset of immune cell types from Satpathy *et al*. [49] (referred to as “Satpathy immune scATAC” in future). We first grouped cells from the study into 8 major cell types based on the original cluster annotations (monocytes, B cells, CD4+ T cells, CD8+ T cells, dendritic cells, progenitor cells, NK cells, and basophils) to obtain reliable peaks for each of the 8 cell types (Methods) [50]. In S-LDSC analysis on this dataset, monocyte and dendritic cell scATAC OCRs displayed a statistically significant enrichment for colocalization with AD risk relative to OCRs for the other immune cell types **(Figs 4C and S2)**.

### Peripheral immune cell types display stronger enrichment for AD-associated regulatory variants compared to brain resident microglia

To investigate the relative roles of brain-resident microglia and immune cells in AD, we sought to compare brain-resident microglial signals and peripheral immune signals directly in an S-LDSC analysis. To do this, we compared CD14+ monocyte OCRs (DNase-seq from ENCODE: ENCFF231SIM, ENCFF000TBP, ENCFF690LNW, ENCFF000TBL) [39] and microglia OCRs (ATAC-seq from Gosselin *et al*.) [51]. DNase-seq and ATAC-seq both measure chromatin accessibility, are highly correlated and retrieve highly similar OCR peaks [52]. Since bulk ATAC-seq from sorted CD14+-monocytes is not readily available, we compared the different assays. Surprisingly, we found that CD14+ monocyte OCRs displayed a significant enrichment relative to microglia OCRs for the Kunkle *et al*. GWAS [8] of diagnosed AD (LDSC coefficient = 2.95e-7,p = 0.019) **(Fig 4D)**. With the Jansen *et al*. [9] GWAS of diagnosed AD and AD-by-proxy, the CD14+ monocyte datasets displayed a higher enrichment coefficient than the microglial dataset; however, the enrichment was not statistically significant (LDSC coefficient = 4.41e-8,p = 0.116) **(S2 Fig)**.

Further, we evaluated enrichment in H3K27ac chromatin immunoprecipitation (H3K27ac ChIP-seq) peaks which, unlike DNase-seq and ATAC-seq defined OCRs, can differentiate between activating and repressive transcription factor binding [53]. We found that H3K27ac ChIP-seq peaks for monocyte are significantly enriched over H3K27ac ChIP-seq peaks for microglia in a direct comparison for two GWAS studies (Jansen *et al*. [9] coefficient = 1.77e-08, p = 0.0038; Kunkle *et al*. [8] coefficient = 7.49e-08, p = 0.019). To assess whether these results were robust to noise in the Roadmap Epigenomics peak calling approach, we reran the analysis with more stringent monocyte H3K27ac ChIP-seq peaks (p<1e-5). Again, we observed similar results (Jansen *et al*. [9] coefficient = 2.02e-08, p = 0.003; Kunkle *et al*. [8] coefficient = 7.49e-08, p = 0.023) suggesting a strong monocyte enrichment over microglia. To evaluate whether these results replicate in other datasets, we also applied this pipeline to another monocyte/macrophage dataset from Alasoo *et al*. 2018 [22] and two other microglia datasets: one from Kosoy *et al*. 2022 [54] and a single nucleus ATAC-seq (snATAC-seq) dataset from Corces *et al* 2020 [20]. In addition, we included more recent and larger GWAS studies from Wightman *et al*. [11] and Bellenguez *et al*. [10]. After correcting for multiple hypotheses across all these tests, we found multiple examples where monocytes displayed stronger enrichment over microglia. In some cases, most notably for the Bellenguez *et al*. [10] GWAS, we instead observed microglia enrichment over monocyte. Additionally, conditional S-LDSC analysis [55] with different deciles of OCR signals from the two cell types also did not suggest a consistent enrichment for one cell type over the other **(S3 Fig)**. While these results make it difficult to conclude which cell type influences predisposition more strongly, our analysis suggests that there is a subset of AD-associated loci that exert their influence on disease predisposition exclusively through peripheral blood CD14+ monocytes and not through brain-resident microglia. For example, a sentinel variant in the Jansen *et al*. [9] GWAS near the ALPK2 locus identified in the Jansen *et al*. [9] GWAS study, rs76726049, lies in a region that is in an OCR in the ENCODE CD14+ monocyte data but not in the Gosselin *et al*. [51] microglia dataset **(Fig 4E)**, suggesting monocyte-specific regulatory effects for this variant. Further interpretation of individual loci is required to interpret which cell types are likely to be primarily impacted.

### CNN classifiers and regression models trained on open chromatin measurements from AD-relevant cell types can accurately predict regulatory activity from sequence

**Fig 5:**
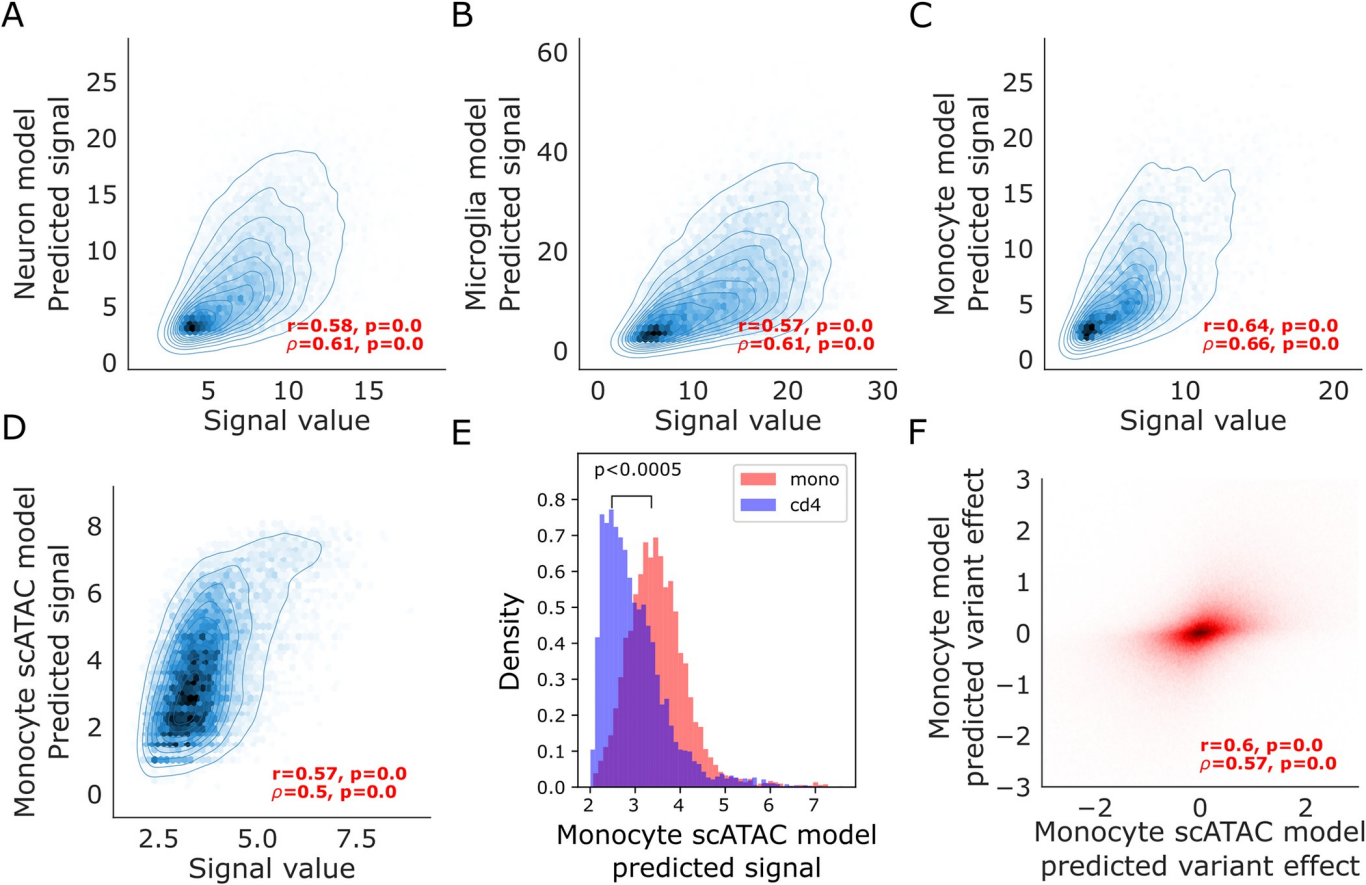
Performance of CNN regression models trained on bulk and single cell ATAC-seq datasets from AD relevant cell types on held out peaks A. Scatter plot of true ATAC-seq signal from the peak caller vs predicted ATAC-seq signal on test set examples (chromosomes 8 and 9) from a CNN regression model trained on open chromatin peaks from the Fullard *et al*. [56] NeuN+ neuronal ATAC-seq dataset **B.** Similar to A. but for a model trained on open chromatin peaks from the Gosselin *et al*. [51] microglia ATAC-seq dataset **C.** Similar to A. and B. but for a model trained on open chromatin peaks from the ENCODE CD14+ monocyte DNase-seq dataset **D.** Scatter plot of true scATAC-seq signal derived from ArchR peak calling on pseudo bulk samples of all monocytes in the scATAC-seq dataset versus predicted signal on test set. (chromosomes 8 and 9) peaks from a CNN regression trained on the monocyte ArchR peaks **E.** Histograms of predicted signal values from a CNN regression model trained on monocyte scATAC-seq peaks from the Satpathy *et al*. [49] dataset on peaks that are exclusively active in monocytes relative to CD4 T cells and peaks that are exclusively active in CD4 T cells relative to monocytes. A Mann- Whitney test was used to test for difference between the means of the predicted signals for the two distributions. p-value from the test is indicated **F.** Scatter plot of predicted variant effects from the bulk ENCODE monocyte trained CNN regression model vs the predicted variant effects from the scATAC monocyte trained CNN regression model for all annotated variants in the genome included in the Tanzi variant set.

Notably, S-LDSC only looks at colocalization between the genetic association signals in GWAS and the genomic regions of interest. To identify functional variants, it is necessary to show that a variant has an impact on the regulatory activity of an OCR. Having identified peripheral monocytes and microglia as strong candidate cell types for influencing AD predisposition in S-LDSC analyses, we trained CNN regression models on the ENCODE CD14+ monocyte and the Gosselin microglia dataset. We conducted model training in a similar fashion to the models trained on the GM12878 DNase-seq dataset (Methods). In addition, we trained a CNN regression model on a putamen neuronal (NeuN+ sorted nuclei) ATAC-seq dataset from Fullard *et al*. [56] (“Fullard NeuN+ ATAC”) since neuron damage is a key consequence of AD. We found that CNN regression models trained on all three of these datasets were able to generalize and predict open chromatin signals accurately on completely held out chromosome 8 and 9 OCRs in each cell type **(Figs 5A–5C and S4)**.

Since we had bulk chromatin accessibility data for only two immune cell types and we would like to predict the effects of AD variants on multiple immune cell types and identify immune cell type-specific AD variant effects, we also trained models using data from cell type clusters from scATAC-seq. Specifically, we applied our CNN regression model training framework on the 8 cell types in the Satpathy *et al*. [49] immune cell scATAC-seq dataset. However, scATAC-seq data presents challenges since the peaks x counts matrix is usually sparse for individual cell types. This sparsity can be introduced by inefficient transposase activity and low cell number for rare cell types that in turn leads to low read coverage in these cell types. Therefore, processing and predicting the signal value in scATAC-seq datasets can be challenging. To obtain robust signals, we first grouped the original cell clusters from Satpathy *et al*. [49] into 8 major cell classes (monocytes, B cells, CD4+ T cells, CD8+ T cells, dendritic cells, progenitor cells, NK cells, and basophils) and performed peak calling using ArchR [50]. Then, we trained 8 different single-task CNN regression models for the 8 cell types, modeling ArchR defined signal at the OCR peak as a function of 500 bp expanded peak summit sequence inputs. Our CNN regression models were able to accurately predict the ArchR signal for all 8 cell type groupings **(Figs 5D and S5)**. We found that correlation between true signal values and predicted signal values for the holdout test dataset were strong but lower than those for bulk datasets (Pearson’s r between 0.52–0.65 and Spearman’s ρ between 0.49–0.59) (**Table 1).** This is to be expected due to the noise and sparsity in scATAC-seq.

**Table 1:** Cell types from the bulk open chromatin and scATAC dataset, number of OCRs, genome coverage, and model performances for these cell types, number of AD-associated variants overlapping OCR peaks and candidate functional AD outlier variants identified by the CNN models. Candidates near the HLA locus are included in a separate column.

Cell type	Num OCR Peaks	Genome Coverage	Validation set r	Validation set ρ	Test set r	Test set ρ	Overlapping AD variants	Shapiro-Wilk Statistic (p-value)	Candidate outlier variants	Candidate HLA locus variants
Bulk ENCODE monocyte	222431	2.4%	0.65	0.67	0.64	0.66	170	0.81 (0.0)	rs636317, rs1010322, rs2526377, rs76726049, rs28834970	rs9270921, rs9271182
Bulk Gosselin microglia	180768	1.3%	0.6	0.64	0.57	0.61	134	0.78 (0.0)	rs636317, rs6064392	rs9271162
Bulk Fullard NeuN+ Putamen	176937	1.2%	0.6	0.63	0.58	0.61	45	0.85 (0.0)	rs395601	None
scATAC Monocytes	163220	2.6%	0.57	0.51	0.57	0.5	150	0.92 (0.0)	rs636317, rs636341, rs28834970	rs9271145
scATAC Dendritic cells	181998	2.9%	0.52	0.49	0.52	0.49	119	0.91 (0.0)	rs636317, rs28834970, rs514049, rs6064392, rs2526377	rs9270920, rs9270921
scATAC Progenitors	193867	3.1%	0.58	0.51	0.58	0.51	101	0.90 (0.0)	rs636317, rs2526377, rs75045569	rs9270921
scATAC Basophil	70593	1.1%	0.65	0.57	0.65	0.57	39	0.92 (0.0)	rs6064392, rs28834970	rs9271171
scATAC Natural Killer cells	100127	1.6%	0.56	0.52	0.56	0.51	64	0.96 (0.0)	rs514049, rs1010322, rs73223431	None
scATAC CD4 T cells	139595	2.2%	0.65	0.59	0.65	0.59	73	0.92 (0.0)	rs514049, rs111278892	rs9270921, rs9271162, rs9271170
scATAC CD8 T cells	128386	2.0%	0.62	0.57	0.63	0.59	70	0.93 (0.0)	rs4575098	rs9270921, rs9271162
scATAC B cells	116993	1.9%	0.53	0.48	0.53	0.49	113	0.97 (0.0)	None	

### scATAC-seq trained CNN models predict cell type-specific signals

Many enhancer variants have effects in only specific cell types and, to capture this, a model needs to be able to distinguish between enhancers active in a cell type and not in another cell type. Our scATAC monocyte model predicted significantly lower scores for CD4+ T cell peaks that are not peaks in monocytes (p<0.0005) **(Fig 5E)**. Model predictions of variant effect from CNNs trained on bulk CD14+ monocytes and CNNs trained on the scATAC monocytes displayed strong correlations (r = 0.6, ρ = 0.57) **(Fig 5F).** Combined, this suggests that our scATAC models are able to predict regulatory activity accurately for a cell type as well as capture signals specific to a cell type.

### CNN classifiers and regression models identify candidate functional variants in AD-associated loci that act through specific cell types

To construct an input set of AD-associated genetic variants, we identified a set of genetic variants that are in linkage disequilibrium (LD r^2^> 0.5) with sentinel genetic variants identified in two AD GWAS, Jansen *et al*. [9] and Kunkle *et al*. [8]. Using this criterion, we constructed a set of 1209 LD-expanded AD-associated variants. For scoring a variant, we propagated two sequences through a model, one centered on the reference allele of the variant and the other centered on the alternate allele of the variant. We propagated each of the 1209 sequence pairs through all 11 models and computed predicted allelic skew by computing the difference between the model output for the reference-allele-carrying sequence from the alternative-allele-carrying sequence. Since CNN models are known to have difficulties generalizing to areas of the input space that are outside of the training distribution [57], we focused the initial interpretation of model scores on only AD-associated genetic variants that overlapped with OCRs in the respective cell type/dataset **(Table 1)**. To construct a null distribution of predicted variant effects, we made predictions for the full set of variants annotated for the human genome [58] that lie in OCRs for a given cell type using the model trained on that cell type. Upon visual inspection, model predictions of variant effect followed a Gaussian-like distribution centered around 0 but with a heavy tail **(S6 Fig)**. We conducted Shapiro-Wilk tests for normality and found that none of the distributions followed a Gaussian distribution **(Table 1)**. For ease of interpretation, however, we used properties of the Gaussian distribution to model variant effect predictions. For each model, we normalized all variant effect scores by dividing them by the standard deviation of allelic skew effect scores for all annotated variants that overlap the respective open chromatin annotations for that model. We did this to mimic a Z-score normalization for a distribution with a mean of 0. From this distribution, we identified outliers by selecting all OCR-overlapping variants that lie at least 2 standard deviations away from the 0 value. We use this cutoff because around 95% of values lie within 2 standard deviations of the mean of a Gaussian distribution. Roughly, this corresponds to a p-value cutoff of 0.05, which is widely used in scientific literature ever since it was described in R.A. Fisher’s tables [59,60]. Notably, the heavy tail of the empirical distribution actually renders this cutoff more stringent than a p-value cutoff of 0.05. To confirm this, we attempted to fit a Laplace distribution to the scores from one of our models by sampling from a Laplace distribution with the same mean and standard deviation. We found that the Laplace-distributed samples were distributed in a way more similar to the data’s distribution than the Gaussian-distributed samples; however, the Laplace distribution was still unable to capture the heavy tail **(S6 Fig)**. Notably, the 2 standard deviation (s.d.) threshold based on a Gaussian distribution corresponds to a 2√2 = 2.82 s.d. threshold based on the Laplace distribution, which is closer to the 4 s.d threshold that represents a Bonferroni-corrected p-value of 0.05. Considering that the Laplace distribution is also unable to account for the heavy tail and for the purposes of simplicity and ease of interpretation, we chose to continue using the 2 s.d. Gaussian threshold. We provide Benjamini Hochberg FDR corrected q-values based on the cumulative distribution function of the Gaussian distribution to correct for multiple hypotheses. However, we note that a future extension would be necessary to use another distribution, such as a Cauchy distribution or a Student’s t distribution with the correct degree of freedom. These distributions have an even heavier tail and can model the distribution of variant effect scores better. For additional interpretation of the small set of identified outlier variants, we relaxed our initial overlap criteria and scored their effects using models for other cell types even if the variant did not overlap an OCR in that cell type.

From the initial analysis, we found 7 OCR-overlapping AD-associated outlier variants that showed predicted effects in CD14+ Monocytes, 3 outlier variants for Microglia, and 1 outlier variant for Neurons. The 7 monocyte outlier variants were identified near the genes MS4A, ABI3, BZRAP-AS1/TSPOAP1, ALPK2, HLA-DRB1, and CLU/PTK2B; the microglia outlier variants were near the genes MS4A, CASS4, and HLA-DRB1; and the neuron variant was near the gene ADAM10. Notably, MS4A, ABI3, PTK2B, and HLA-DRB1 are highly expressed in both microglia [61] and monocytes/macrophages, and CASS4 is highly expressed in microglia [61]. The gene near the neuron outlier variant ADAM10 is a metalloproteinase that forms a major component of α-secretase involved in processing of amyloid β (Aβ) in neurons [62]. A complete list of all identified outlier variants can be found in **Table 2**. Of the 7 monocyte candidates, the 4 variants near ALPK2 (rs76726049), CLU/PTK2B (rs28834970), and HLA-DRB1 (rs9270921, rs9271182) did not overlap a microglia peak, suggesting an effect exclusive to peripheral immune monocytes and not brain resident microglia.

**Table 2:** non-HLA candidate functional variants identified by models for different cell types.

Variant	Closest Gene	Potentially Disrupted Regulatory Motifs	Lead/LD	Risk allele	Non-risk allele	Z-Score (Risk -NonRisk)	FDR q-value	Jansen finemapping PIP (In credible set Y/N)	Kunkle finemapping PIP (In credible set Y/N)	Corces *et al*. outlier [20]
**ENCODE CD14+ Monocytes (Bulk)**										
rs636317	MS4A6A	CTCF, E2A, GR, RXRA, TAL1, YY1	LD	T	C	-6.3	2.44 × 10^−8^	8.25 × 10^−4^ (N)	0.004 (Y)	microglia
rs1010322	ABI3	AP-4,LF-A1	LD	C	G	3.53	7.06 × 10^−3^	0.003 (N)	No peak	
rs2526377	BZRAP-AS1	Irf, MYC, Smc3, SP1, Sp4, Spz1, ZNF219, Zfp281, Zfp740	LD	A	G	3.89	2.75 × 10^−3^	0.03 (Y)	No peak	microglia
rs28834970	CLU/PTK2B	CEBPA, CEBPB, CEBPD, Hsf, Stat, p300	LD	C	T	3.53	7.06 × 10^−3^	0.219 (Y)	0.234 (Y)	
**Gosselin Microglia (Bulk)**										
rs636317	MS4A6A	CTCF, E2A, GR, RXRA, Rad21, SMC3, TAL1, YY1	LD	T	C	-10.16	0	8.25 × 10^−4^ (N)	0.004 (Y)	microglia
rs6064392	CASS4	ATF3	LD	G	T	3.74	5.4 × 10^−3^	0.029 (Y)	No peak	
**Fullard NeuN+ Putamen (Bulk)**										
**scATAC Monocytes**										
rs636317	MS4A6A	CTCF, E2A, GR, RXRA, Rad21, SMC3, TAL1, YY1	LD	T	C	-5.39	1.0 × 10^−5^	8.25 × 10^−4^ (N)	0.004 (Y)	microglia
rs636341	MS4A6A	CTCF, CEBPB, HMG-IY, NF-kappaB, RXRA, Rad21, YY1	LD	C	A	2.23	6.2 × 10^−1^	7.22 × 10^−4^	0.005 (Y)	
rs28834970	CLU/PTK2B	CEBPA, CEBPB, CEBPD, Hsf, Stat, p300	LD	C	T	3.31	6.7 × 10^−2^	0.219 (Y)	0.234 (Y)	
**scATAC Dendritic Cells**										
rs636317	MS4A6A	CTCF, E2A, GR, RXRA, Rad21, SMC3, TAL1, YY1	LD	T	C	-4.42	1.13 × 10^−3^	8.25 × 10^−4^ (N)	0.004 (Y)	microglia, neuron, astrocyte, oligo
rs28834970	PTK2B	CEBPA, CEBPB, CEBPD, Hsf, Stat, p300	LD	C	T	3.2	2.6 × 10^−2^	0.219 (Y)	0.234 (Y)	
rs514049	ADAM10	ATF3, HEY1	LD	C	A	3.60	1.2 × 10^−2^	7.88 × 10^−6^ (N)	No peak	
rs6064392	CASS4	ATF3	LD	G	T	3.64	1.2 × 10^−2^	0.029 (Y)	No peak	
rs2526377	BZRAP-AS1	Irf, MYC, Smc3, SP1, Sp4, Spz1, ZNF219, Zfp281, Zfp740	LD	A	G	3.40	1.9 × 10^−2^	0.03 (Y)	No peak	microglia, neuron, astrocyte, oligo
**scATAC Progenitors**										
rs636317	MS4A6A	CTCF, E2A, GR, RXRA, Rad21, SMC3, TAL1, YY1	LD	T	C	-6.98	2.96 × 10^−10^	8.25 × 10^−4^ (N)	0.004 (Y)	
rs2526377	BZRAP1	Irf, MYC, Smc3, SP1, Sp4, Spz1, ZNF219, Zfp281, Zfp740	LD	A	G	3.10	6.3 × 10^−2^	0.03 (Y)	No peak	
rs75045569	EPHA1	EBF	LD	T	G	-2.15	7.68 × 10^−1^	0.001 (N)	0.036 (Y)	microglia, neuron, astrocyte, oligo
**scATAC Basophil**										
rs636317	MS4A6A	CTCF, E2A, GR, RXRA, Rad21, SMC3, TAL1, YY1	LD	T	C	-5.39	1.0 × 10^−5^	8.25 × 10^−4^ (N)	0.004 (Y)	
rs636341	MS4A6A	CTCF, CEBPB, HMG-IY, NF-kappaB, RXRA, Rad21, YY1	LD	C	A	2.23	6.2 × 10^−1^	7.22 × 10^−4^	0.005 (Y)	
rs2526377	BZRAP1	Irf, MYC, Smc3, SP1, Sp4, Spz1, ZNF219, Zfp281, Zfp740	LD	A	G	2.38	4.9 × 10^−1^	0.03 (Y)	No peak	
rs6064392	CASS4	ATF3	LD	G	T	2.59	3.4 × 10^−1^	0.029 (Y)	No peak	
rs28834970	CLU/PTK2B	CEBPA, CEBPB, CEBPD, Hsf, Stat, p300	LD	C	T	3.31	6.7 × 10^−2^	0.219 (Y)	0.234 (Y)	
**scATAC Natural Killer Cells**										
rs514049	ADAM10	ATF3, HEY1	LD	C	A	3.01	5.3 × 10^−2^	7.88 × 10^−6^ (N)	No peak	microglia
rs1010322	ABI3	AP-4,LF-A1	LD	C	G	3.01	5.3 × 10^−2^	0.003 (N)	No peak	microglia, neuron, astrocyte, oligo
rs73223431	CLU/PTK2B	Brachyury, CEBP, Eomes	Lead	T	C	-2.50	1.7 × 10^−1^	0.219 (Y)	0.234 (Y)	microglia
**scATAC CD4 T cells**										
rs514049	ADAM10	ATF3, HEY1	LD	C	A	3.53	1.4 × 10^−2^	7.88 × 10^−6^ (N)	No peak	
rs111278892	ABCA7	GATA3	Lead	G	C	2.75	8.5 × 10^−2^	0.569 (Y)	0.001 (N)	
**scATAC CD8 T cells**										
rs4575098	ADAMTS4	Arid5a, Cdx2_2, DMRT2, DMRT3, Dbx1, Foxj2_2, Hoxa10, Hoxb13, Hoxb9, Hoxd10, Hoxd8, NF-Y_known1, Ncx_2, Pou1f1_1, Pou2f2_known2, Pou3f3, SP1_disc1, Zfp187	Lead	A	G	-2.16	8.56 × 10^−1^	0.72 (Y)	No peak	
**scATAC B cells**										
rs6064392	CASS4	ATF3	LD	G	T	3.22	1.7 × 10^−2^	0.029 (Y)	No peak	
rs28834970	CLU/PTK2B	CEBPA, CEBPB, CEBPD, Hsf, Stat, p300	LD	C	T	3.26	1.7 × 10^−2^	0.219 (Y)	0.234 (Y)	

Our bulk ENCODE CD14+ monocyte model identified rs28834970 (-3.52 s.d. from 0; FDR q = 7.06 × 10^−3^) near the CLU and PTK2B genes as an outlier. Notably, our scATAC-seq models trained on monocytes (-3.31 s.d. from 0, FDR q = 6.7 × 10^−2^,nominal p-value = 9.3 × 10^−4^), dendritic cells (-3.2 s.d. from 0, FDR q = 2.6 × 10^−2^) **(S7 Fig)**, B cells (-3.26 s.d. from 0, FDR q = 1.7 × 10^−2^), and basophils (-3.26 s.d. from 0, FDR q = 6.7 × 10^−2^,nominal p-value = 9.3 × 10^−4^) all predicted this variant as an outlier, indicating that the effect of this variant may be shared across these peripheral immune cell types. This is in line with an earlier study which indicated that this variant alters a CEBP binding site, which leads to altered accessibility of the regulatory element and altered expression of the nearby gene PTK2B in macrophages [22]. Our models predicted the reference allele (also the risk allele) T for rs28834970 to have a decreased regulatory activity in these cell types. Further, this variant is part of a finemapped 95% credible set in both the Jansen *et al*. [9] (PIP = 0.219) and the Kunkle *et al*. [8] GWAS (PIP = 0.234) and has a significant eQTL association with the PTK2B gene in LCLs (p = 1.22e-07) [63]. Even though the variant did not overlap an OCR in the Gosselin microglia dataset, the model score for this variant was strong (-2.05 s.d. from 0). Interpretation on the monocyte model using DeepSHAP [64] on the rs28834970 sequence pair assigned strong positive contribution scores to the C allele and negative contribution scores to the T allele **(S8 Fig)**. Additionally, the model assigned high contribution scores to nearby nucleotides that form part of a CEBP motif. CEBP factors are expressed highly in both microglia and peripheral blood cell types. Notably, in differential motif enrichment analysis using Centrimo [65], 5 CEBP factors including CEBPα (E-value = 4.8 × 10^−85^),CEBPβ (E-value = 5 × 10^−802^),CEBPγ (E-value motif 1 = 2.4 × 10^−885^,E-value motif 2 = 1 × 10^−140^),δ (3.7 × 10^−147^), and CEBPε (7.0 × 10^−937^) were centrally enriched in ENCODE monocyte OCRs relative to the Gosselin *et al*. [51] Microglia OCRs, suggesting peripheral immune-specific regulation mediated by these factors.

**Fig 6:**
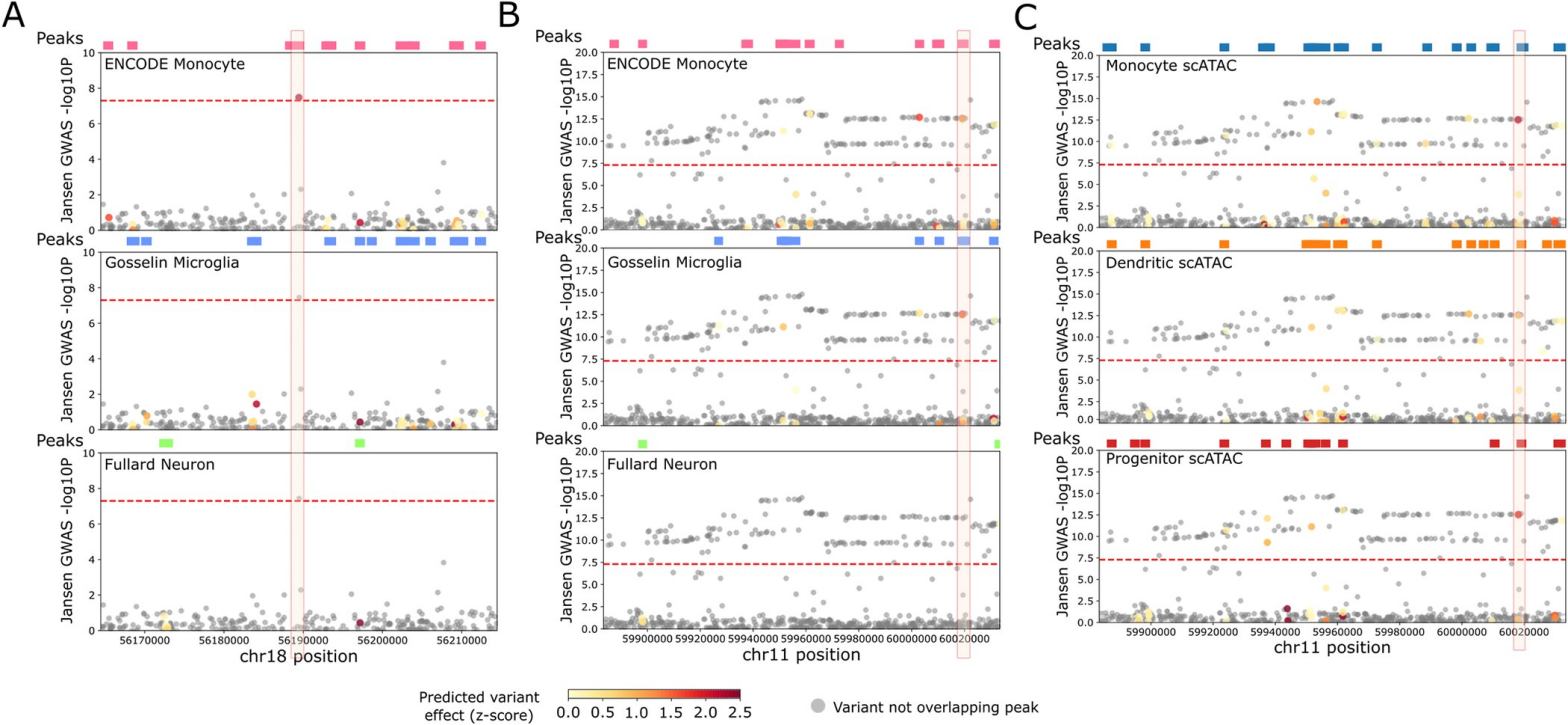
Candidate functional variants identified from models display effects in immune cell types which in some cases, are specific to peripheral immune cell types compared to brain resident microglia A. Genome browser visualization of the AD association region in the Jansen *et al*. [9] GWAS at the locus containing the ALPK2 gene. Tracks include open chromatin annotations from the ENCODE monocyte DNase-seq dataset, the Gosselin *et al*. [51] microglia ATAC-seq dataset, and the Fullard *et al*. [56] NeuN+ ATAC-seq dataset as well as model predictions from the corresponding models trained on that cell type’s dataset. Specifically, the track below each peak annotation track shows the Jansen *et al*. [9] GWAS p-value (-log10 transformed) of each variant in the shown window and the variants are colored by the strength of predicted variant effect from the CNN regression model trained on that corresponding cell type’s dataset. The red highlighted box indicates the location of the sentinel variant at this locus (rs76726049), which overlaps a monocyte peak and is also predicted to have a strong effect on the regulatory activity of that peak from the ENCODE monocyte CNN regression model. Variants that do not overlap open chromatin peaks in the corresponding cell type’s dataset were not scored using the models and are shown in grey. **B.** Similar to A. but for the AD-associated region near the MS4A locus. The red highlighted box indicates the location of rs636317 which overlaps both a microglia and a monocyte peak and displays a strong predicted effect from both the monocyte and microglia trained CNN models. **C.** Similar to B. but includes annotations from the scATAC- seq dataset as well as model predictions from the CNN regressions trained on the Satpathy *et al*. [49] scATAC-seq cell types. Shown are scATAC-seq based annotations and predictions for monocyte, dendritic cells, and progenitors but predictions for other cell types were also made. These three cell types displayed peak overlaps with rs636317 in the scATAC-seq dataset and also a significant predicted effect for this variant on regulatory activity of the corresponding overlapping peak.

Matching the results of S-LDSC analysis, there were several variants where the CNN models predicted a peripheral immune-specific effect on gene regulation. rs76726049 is a sentinel variant identified in the Jansen *et al*. [9] GWAS near the ALPK2 gene **(Fig 6A)**. This variant displayed a large predicted effect (2.3 s.d. from 0, nominal p-value = 1.97 × 10^−2^) even though it wasn’t significant after multiple hypothesis correction (FDR q = 0.23) from our ENCODE CD14+ monocyte model and no other model. In contrast, the variant does not overlap an OCR in the Gosselin *et al*. [51] microglia dataset and the Gosselin microglia trained model predicted a weak effect for this variant (0.74 s.d. from 0) **(S7 Fig)**. In addition, the monocyte classifier predicted a very small non-significant effect for this variant (0.18 s.d. from 0). Combined, this suggests that rs76726049 may influence AD predisposition by subtly altering regulatory activity in peripheral monocytes and not in microglia. We conducted model interpretation for rs76726049 sequence pair using DeepSHAP [64] on the monocyte model, and we found that the model attends strongly to the index position of the variant for the reference-allele-carrying sequence but does not do so for the alternate-allele-carrying sequence **(S9 Fig)**. Additionally, the model focuses on adjacent nucleotides on the reference-allele-carrying sequence, suggesting that the surrounding sequence pattern that is important for prediction of monocyte regulatory elements is disrupted by the alternate allele of the sequence. Motif search near the variant revealed Znf341 and Znf530 motifs overlapping these adjacent nucleotides that the model focuses on, suggesting a potential disruption of Znf341 or Znf530 binding by this variant in monocytes. Notably, in differential motif enrichment analysis using Centrimo [65], both Znf341 (E = 1.3e-103) and Znf530 (E = 3.2e-407) motifs are enriched in the ENCODE monocyte OCRs relative to the Gosselin *et al*. [51] microglia OCRs. This variant is the only significant variant at this locus in the Jansen *et al*. [9] GWAS and hence is most likely the causal variant. Finemapping on the Jansen *et al*. [9] GWAS at this locus confirmed this result (PIP = 0.997). The identification of the peripheral immune-specific effect of rs76726049 demonstrates the ability of our models to identify candidate variants influencing AD predisposition and the subtle differences between the similar regulatory element landscapes of peripheral immune cell types and brain-resident microglia.

The monocyte and microglia models both predicted rs636317 near the MS4A locus as an outlier **(Fig 6B)**. This variant has previously been reported to display allelic imbalance on the overlapping ATAC-seq peak in myeloid cells [18] and has been predicted by another CNN model to have a strong effect in microglia [20]. Additionally, it is part of a 95% credible set in the Kunkle *et al*. [8] study (PIP = 0.004) and has significant eQTL association with the MS4A6A gene in whole blood (p = 1.22e-07, [66]). Our models predict that the AD risk allele led to lower regulatory activity, and this direction of effect is consistent with the Novikova *et al*. [18] study that showed that the risk allele disrupts a CTCF transcription factor binding site to decrease regulatory activity. rs636317 does not overlap an OCR in the Fullard *et al*. [56] neuron model; however, it displayed a strong predicted effect **(S7 Fig)**, leaving open the possibility that the variant also has an effect in neurons. Our scATAC-seq-trained models also called rs636317 as an outlier for monocytes, dendritic cells, and progenitor cell types **(Fig 6C)**. Similar to the bulk models, the scATAC-trained models on monocytes, dendritic cells, and progenitors predicted a decrease in regulatory activity for the AD risk allele.

Near the ADAM10 gene, our dendritic cell (3.59 s.d. from 0, FDR q = 1.2 × 10^−2^), natural killer cell (3.01 s.d. from 0, FDR q = 5.3 × 10^−2^, nominal p-value = 2.5 × 10^−3^), and CD4 T cell (3.53 s.d. from 0, FDR q = 1.4 × 10^−2^) scATAC-seq-trained models identified rs514049 as an outlier. Our bulk neuron model identified a different outlier variant rs395601 near ADAM10 (-2.49 s.d. from 0, FDR q = 2.2 × 10^−1^,nominal p-value = 1.3 × 10^−2^), which suggests that different variants may contribute to immune-specific and brain-specific effects of ADAM10 gene expression on AD. Notably, ADAM10 displays high expression in peripheral immune cell types [61] as well as neurons, where it is involved in Aβ processing.

### Pre-training MPRA-based models with DNase-seq-trained models enables MPRA-based models to achieve good performance with less MPRA data

While our models trained directly on the MPRA data worked better than our DNase-seq trained models on the task of predicting MPRA activity, our MPRA-trained models used an MPRA with over 70,000 sequences, and generating such large MPRAs for most non-LCL non-cancerous cell types is infeasible due to challenges involved in culturing non-LCL non-cancerous cells. Therefore, we trained models on smaller subsets of the MPRA training data to identify how many samples would be needed to train good MPRA models. As expected, we found that reducing training set size led to lower generalization performance on MPRA test set sequences **(S10 Fig)**. We therefore evaluated model performances in a transfer learning framework where we pre-trained models on the DNase-seq data and fine-tuned them on a subset of the MPRA data. For pre-training, we used a CNN regression model trained on LCL DNase-seq-defined OCRs with GC-content-matched negative sequences that were labeled as having 0 signal. We found that this model generalized well to DNase-seq-defined OCRs on set chromosomes that were not used for training (Pearson’s r = 0.794 for DNase-seq defined OCRs, Spearman’s ρ = 0.766). Then, we fine-tuned this DNase-seq-pretrained model for MPRA prediction on the same subsets of the MPRA training dataset that were used to create the models with subsets of the MPRA data. We found that fine-tuning the model on a quarter of the MPRA training dataset led to reasonable performance on held out sequences (r = 0.393, ρ = 0.547). Strikingly, we found that the transfer learning model fine-tuned on that quarter of the MPRA training set performed much better at predicting a completely held out MPRA test set (r = 0.394, ρ = 0.381) than both the MPRA-only model trained on one quarter of the MPRA training set (r = 0.297, ρ = 0.227) and the model trained on DNase-seq without fine-tuning (r = 0.304,ρ = 0.22) **(S11 Fig)**. We confirmed that these results were not influenced by which of the samples went into the quarter set of samples by fine-tuning 3 additional models on the remaining non-overlapping quarters of the MPRA training set **(S10 Fig)**. Summarizing across all 4 transfer learning models, we achieved a mean r = 0.371 (SD = 0.038) and mean ρ = 0.343 (SD = 0.039). Additionally, all 4 transfer learning models outperformed a DNase-only model at prediction of expression and variant effects in the MPRA test set **(S10 and S11 Figs)**. This suggests that deriving information from larger datasets that assay open chromatin and fine-tuning models on MPRA data can improve prediction performance for sparser MPRAs. Since LCL lines are also derived from B-cells, we also sought to predict the effects of AD-associated variants to identify outliers. Interestingly, we were able to identify effects of only variants in the HLA locus with our transfer learning models. This locus is hard to interpret because of its exceptional nature of linkage and variation [67]. Nevertheless, the subset of AD SNPs that overlap OCR peaks in both LCL and AD-enriched cell types can be further studied using our LCL models. Overall, our transfer learning approach is a proof–of-concept and, in future work, it would be important to build transfer learning models on *in vivo* MPRA data [68] from the relevant cell types and cell states for better interpretation of FAD-SNP effects.

## Discussion

Computational genomic approaches have been valuable for linking candidate disease-associated genetic variants to regulatory function in specific tissues and cell types. However, these inferences may be confounded when compared across cell types that are highly similar. Here, we propose a computational framework which enables prediction of variant effects on regulatory activity in disease-relevant cell types. First, we use stratified LD score regression (S-LDSC) to directly compare GWAS association data with open chromatin peaks in multiple cell types. Next, for the implicated cell types, we train regression convolutional neural networks that are able to predict the cell type-specific effect of candidate genetic variants. We are also able to validate the increased sensitivity of our models using MPRA data.

Using this framework, we systematically compare the regulatory impact of genetic variants on multiple neural and immune cell types that have been implicated in the predisposition to AD, some of which are highly similar. Surprisingly, both S-LDSC and the regression CNN approach suggest strong roles for peripheral monocytes as well as brain-resident microglia. First, we find that both blood cell types and microglia are enriched for LOAD-associated genetic variants relative to other tissues. We also find that for some GWASs, blood myeloid cell OCRs display a stronger enrichment than microglia, the brain-resident myeloid cell type. This suggests that, while many AD-associated variants lie in regulatory elements shared between blood and brain immune cell types, a significant subset lie in regulatory elements that are active in only blood immune cell types. Second, several of the candidate FAD-SNPs we identify are predicted by CNN regression models to have a stronger impact on gene regulation in CD14+-monocytes than microglia, are part of finemapped credible sets derived from AD GWAS and have relevant eQTL associations in the corresponding cell types. While multiple groups have sought to understand the function of regulatory variants in microglia [18,20], our results indicate both a shared mechanism of AD progression between microglia and blood myeloid cells, and certain exclusive mechanisms of AD progression specifically through blood myeloid cells such as CD14+-monocytes. This necessitates a strong focus on peripheral blood immune cell types in the future study of AD-associated regulatory variant effects.

We acknowledge that the variants unique to CD14+-monocytes may act only in specific microglial states such as activated microglial states associated with AD related inflammation that our microglia datasets might not have captured. Therefore, future research on the function of regulatory AD-associated variants needs to include not only models of inactivated microglia but also other microglial states, variety of which have been observed in the AD brain [69,70]. Comparing between immune cell types using scATAC-seq data, we find monocytes and dendritic cells are enriched for AD regulatory genetic variants suggesting that a stronger focus on these cell types is also needed in studies of AD regulatory genetic variants. However, this does not support the exclusion of other immune cell types since our previous analysis suggests immune cell types are enriched overall compared to other non-immune tissues and cell types. Further, it is possible that a subset of AD-associated variants may act only in stimulated blood myeloid cell states similar to microglia. For example, future studies could include stimulated blood myeloid cell states.

Using our models, we identified both known and novel variants that are predicted to disrupt regulatory activity in the relevant cell types identified in S-LDSC analysis. For example, our models were able to identify a peripheral immune-specific effect of rs28834970 in the CLU/PTK2B locus which recapitulates its previously reported role in altering chromatin accessibility. Model interpretation revealed that this variant disrupts nearby binding sites for CEBP factors which are expressed in both microglia and monocytes. Notably, CEBP motifs are more enriched in peripheral immune OCRs relative to microglia OCRs suggesting certain peripheral immune specific mechanisms. Future work investigating the peripheral immune-specific roles of the CLU/PTK2B, rs28834970, and its disrupted transcription factors is required. We also found rs76726049, a variant near the ALPK2 gene as having a CD-14+ monocyte-specific effect relative to microglia from our bulk open chromatin- trained models (**Fig 6**). While this variant displayed a strong effect (2.3 s.d. from 0), it did not pass our multiple hypothesis correction threshold based on a Gaussian distribution. We note that the Gaussian based threshold is more stringent than required and, in the future, a more appropriate distribution which can better fit the empirical distribution is required. Future work can investigate whether this variant is significant given the appropriate scoring distribution. Model interpretation using DeepSHAP [64] suggested that Znf341 and Znf530 transcription factor binding sites may potentially be disrupted by this variant. Notably, both Znf341 and Znf530 motifs are centrally enriched in the monocyte OCRs relative to the microglia OCRs. In addition, Znf341 and Znf530 are highly expressed in macrophages and monocytes [61] and our analysis suggests that disruption of their binding by rs76726049 could alter monocyte function and influence AD predisposition. The interplay between this variant, Znf transcription factors, and their effects on ALPK2 expression in influencing AD predisposition needs to be studied. This variant did not overlap a monocyte scATAC-seq peak and did not show a strong effect in the monocyte scATAC-seq-trained model. This could be due to low read coverage in monocytes in the scATAC-seq dataset rather than an absence of a regulatory element. Notably, this variant overlapped a scATAC-seq peak identified in the closely clustered dendritic cell population and displayed a moderate predicted effect in the dendritic cell model (1.48 s.d from 0), suggesting that it may not be a false positive based on the bulk CD14+ monocyte model. Since sequencing depth can lead to low peak numbers and sparsity in certain cell types in scATAC-seq, this leads to a decrease in the overall number of variants that can be effectively scored by these models. Additionally, artifacts associated with suboptimal clustering can affect downstream analyses. Therefore, our scATAC-seq models may have a high false negative rate for variant effects. Models trained on deeper and less sparse scATAC-seq datasets will reduce the rate of false negatives.

Genetic variants associated with complex diseases can have relatively subtle effects on regulatory activity. Therefore, it is important that computational approaches that aim to predict variant effects can capture these subtle effects. In our framework, we hypothesized that modeling quantitative signals from regulatory assays could improve variant effect prediction performance compared to the commonly trained binary classifiers in the literature. We show that models trained on separate open chromatin measurements (DNase-seq/ATAC-seq) that solely use inherent genetic variation across the genome reference instead of being explicitly fed in sequences with reference and alternate alleles of variants perform comparably well at predicting the direction of variant effects compared to the models trained directly on MPRA data. As hypothesized, we also found that regression models perform better than our classifiers as well as the Sei model [46] at predicting the direction of variant effects in MPRA. Although further hyperparameter tuning of the CNN classifier could potentially bring performance on par with the CNN regression, the minimally tuned regression models are able to predict the subtle differences in regulatory activity induced by many common variants better than classifiers. Therefore, modeling quantitative OCR signals using regression models instead of relying on a binary label which discards continuous information can prove useful in future modeling. In the future, as MPRA datasets become larger and more robust to technical noise, this difference in performance between classifiers and regression models can be better explored.

We rely on MPRA data for validation since it is not influenced by LD as much as other datasets that have been used for variant effect validation such as eQTL data [29,30,71,72], caQTL data, or GWAS catalog membership. To our knowledge, only two studies have validated variant effect predictions from machine learning models directly on MPRA data [44,45], which is not confounded by LD. The MPRA-Dragonn study [44] trained CNN models directly on the Sharpr-MPRA dataset [73] in the K562 cell line and validated variant effect predictions on a separate MPRA dataset [74] in K562 that identified 40 expression modulating variants. The authors reported that a model trained on MPRA in the same context was able to predict the direction of effect for 32 of 40 variants correctly. Since the sample size in this MPRA is small, validating model predictions in much larger MPRA datasets like the Tewhey *et al*. [38] dataset we used is valuable for demonstrating reliability of model predictions. Hoffman *et al*. [45] addressed this issue by successfully validating model predictions in a larger MPRA dataset. As opposed to the commonly trained classifier models in the literature, Hoffman *et al*. [45] used regression to directly model quantitative signals from open chromatin and histone ChIP-seq experiments as a function of the sequence input and were able to show that these models are able to predict MPRA allelic effects reasonably. However, they did not perform rigorous comparisons against classifiers or models trained directly on MPRA data. We conduct a more rigorous extension of this previous study by training models directly on a subset of the MPRA dataset and comparing performance. We also train both classifiers and regression models on open chromatin data and compare performances with these MPRA-trained models.

We show that models trained on DNase-seq display moderate performance on predicting expression in an MPRA. However, these DNase-seq trained models underperform a MPRA-trained model. MPRA datasets are inherently noisy with low replicate concordance, and hence, it is possible that this difference in performance may be due to the fact that the MPRA-trained model is overfitting to noise in the MPRA data. In addition, MPRA is primarily an episomal assay whereas DNase-seq and ATAC-seq assay chromosomal DNA directly. Further, open chromatin regions sometimes do not function as enhancers or promoters, and this contaminates the training set for open chromatin trained models. All of these factors could be contributing to the drop in performance for both the prediction of expression and variant effect and future work could explore the specific contribution of each factor.

Interestingly, we found that using transfer learning improves performance at predicting expression in MPRA. A model pre-trained on DNase-seq data and fine-tuned on a subset of the MPRA data performed better than models trained solely on DNase-seq or solely on MPRA data. These results suggest that sharing of information and training across assays can help better predict MPRA regulatory activity. For primary tissues, open chromatin datasets are more readily available than MPRA datasets, and training models on open chromatin can help better design MPRA assays. Further, fine-tuning open chromatin pre-trained models on small MPRA datasets can in turn better optimize design of future MPRA experiments. In this way, when there are experimental constraints, it may be possible to attain accurate models of MPRA regulatory activity with a much smaller number of MPRA sequences. This approach could be used to combine open chromatin data and data from a small MPRA to design another small MPRA that would contain likely active regulatory elements, enabling active regulatory elements to be identified without the need for large numbers of cells or especially deep sequencing. We note that using data from LCLs to train these models isn’t sufficient to investigate the function of many disease-associated variants since they may only act *in vivo* in certain cell types or states. Further, since LCLs are derived from peripheral blood lymphocytes, this may also bias the conclusions in favor of the peripheral immune cell types in the case of AD. In future work, this approach could be paired with *in vivo* MPRA [68] to investigate variant effects in whole animals.

Our model predictions of variant effect in AD can aid in the prioritization of variants for experimental assays or could serve as additional sources of information that help validate experimental results. In the former case, we foresee that variants identified by our models could be prioritized for MPRA experiments in primary microglial or peripheral myeloid cells for direct assaying of their regulatory effects. This would be useful when the number of variants/sequences that can be assayed in the experiment is limited due to experimental constraint. Previous MPRAs have largely been conducted in cell lines that are easy to manipulate and allow for assaying of a large number of sequences. Since such cell lines contain chromosomal aberrations and are not especially representative of any primary tissue in a behaving individual, it is important to adapt MPRA experiments to primary tissues. This has proven challenging in the past ([75]), and machine learning models can help prioritize variants for these assays especially in cases when the number of oligonucleotides that can be assayed is limited. In addition, when large MPRA datasets do become available, our model predictions can serve as auxiliary sources of information that can help validate the measurements of variant effects in future MPRA experiments.

One limitation of our models is that they do not take into account enhancer-gene linking data. In most cases, we use the nearest gene as a proxy for which gene an enhancer may be regulating [76]. However, enhancers are known to interact with and regulate even distant genes. Therefore, some of our candidate variants may actually have an effect on a distal gene. Augmenting our model predictions with better cell type-specific enhancer promoter interactome maps could enable identification of the genes which are affected by our candidate functional variants. Further, training on longer contexts [77] using attention-based architectures could help address this issue. Additionally, training on individual genomes instead of training on the reference genome could also be helpful for improving model predictions of variant effect. Further, it would be important to identify the correct null distribution to identify outlier variants. We have used the Gaussian for simplicity and tested a Laplace distribution that has a heavier tail against the distribution of our scores. However, both these distributions failed to capture the heavy tail, and fitting a Cauchy or Student’s t distribution could be useful in future studies.

Overall, our framework enables cell type targeted studies of variants associated with specific diseases. As opposed to training on hundreds of cell types or tissues [29,30,71,72], we focus on specific cell types/tissues that are identified in S-LDSC analysis to be enriched for disease relevance. We then use individual models targeted on disease relevant cell types/tissues to identify variants influencing AD predisposition as well as their associated regulatory elements and cell types. We rigorously validate our models on experimentally measured variant effects and make our library of models and model predictions available to the community to study the effects of variants associated with AD as well as other immune and neurological disorders. We anticipate that our machine learning approach to identify candidate variants with cell type-specific effects on disease as well as our finding that variants the peripheral immune system may bring about AD will provide the foundation of future investigation of gene regulatory mechanisms involved in the earliest stages of AD.

## Materials and methods

### Processing GM12878 open chromatin data and training CNN models on GM12878 OCRs

We reprocessed all the bulk open chromatin datasets and called irreproducible discovery rate (IDR) [78] reproducible peaks using the ENCODE ATAC-seq processing pipeline (https://github.com/ENCODE-DCC/atac-seq-pipeline), which filters low quality reads, maps reads using bowtie2 [79] to the hg38 reference genome, calls peaks using MACS2 [80], filters peaks in regions for which reliable read mapping may not be achievable [81], and assesses which peaks are reproducible using IDR. We collected 5 GM12878 DNase-seq datasets from the ENCODE data portal (Accession IDs: ENCFF057JYL, ENCFF363MQH, ENCFF716JJU, ENCFF855ODQ, ENCFF903PJT) [39,40]. Then, we obtained IDR reproducible peaks for GM12878 by treating each dataset as a biological replicate and running the pipeline on the group of datasets. We used default parameters for DNase-seq data. Since two of the replicates were deeper than the others, we used the “conservative set,” which consisted of the IDR reproducible peaks across those two replicates, as the final peak set. The conservative set is the smaller set out of the IDR reproducible peak set called across replicates and the IDR reproducible peak set called across pooled pseudo-replicates. Overall, we were able to get 110,412 peaks to train, validate, and test CNN models.

### Training regression models on GM12878 DNase-seq data

To get training data for the CNN models, we took each peak summit coordinate, expanded it to 1000 bp by extending equally in both directions and obtained the underlying sequence in the 1000 bp regions from the hg19 reference genome. To enable evaluation of model performance, we split these sequences into training, validation, and test sets by holding out all sequences derived from chromosome 4 for the validation set and all sequences derived from chromosomes 8 and 9 for the test set. We used this approach to mimic the test set used by DeepSea [29] and Sei [46]. Further, chr4 is one of the longer chromosomes, so we chose it to obtain a reasonably sized validation set that did not restrict the number of samples in the training/test samples but was still reliable for robust hyperparameter tuning. To train the CNN models, we constructed one-hot encoded arrays (1000x4-sized matrices) for the training set sequences to facilitate input into CNNs with one-dimensional (1D) convolution units in the first layer. Our CNN regression models contained one or more convolutional layers that followed the first convolutional layer. These convolutional layers were followed by a single max pooling layer with dropout regularization, and then a fully connected layer with sigmoid activation functions. This sigmoid layer was followed by a single output unit with no activation. For the regression models, we used stochastic gradient descent (SGD) to minimize the mean squared error (MSE) between this output and the signal value output from the ENCODE pipeline associated with each training peak/sequence. To select the best-performing architecture and hyperparameters, we evaluated model performance on the chromosome 4 held-out sequences and chose the best-performing model in terms of MSE. We fixed the number of filters in the first convolution layer to 1000 with a filter size of 8 and a stride of 1. We varied the number of convolution layers (2 or 3) following the first layer, the number of filters (filter size 8, stride 1) in these convolution layers (200 or 300), the dropout probability (0, 0.1, 0.5, or 0.8) for the max pooling layer (pooling size 13, stride = 13) after the final convolution layer, the number of units in the sigmoid layer (100 or 200) that came after the max pooling layer, and the learning rate (0.1, or 0.01) and batch size (30 or 100) for SGD. We trained each model for 100 passes through the training set (or “epochs”). We report the test set performance of the model with the best-performance on the validation set based on mean squared error. We used Keras v2.2.4 with a Theano [82] backend to implement our models and trained them on graphics processing units (GPUs). Since Theano is now deprecated, we also provide tensorflow [83] -converted model files in our data upload to facilitate future work.

### Training classifiers on GM12878 DNase-seq data

To train the CNN classifiers, we obtained negative examples of background sequences that do not underlie GM12878 OCRs. Since models of open chromatin have been shown to learn GC content profiles in sequences, we chose to use negative example sequences that matched the G+C-content of the positive example sequences. To achieve this, we used BiasAway [84,85] to generate a set of negative sequences and associated coordinates in hg19 that matched the G+C-content distribution of our training set sequences. In order to remove false negative sequences before training, we then used bedtools subtract [86] to remove sequences in this negative set that overlapped peaks in each of the individual GM12878 DNase-seq replicates, and in the IDR optimal and conservative sets output by the ENCODE pipeline. We constructed a superset of positive and negative sequences in order to train the model. Again, we held out sequences on chromosome 4 as a validation set and sequences on chromosomes 8 and 9 as a test set. We set up the CNN classifiers similar to the regression CNN models with an initial 1D convolution layer followed by one or more additional 1D convolution layers and a max pooling layer with dropout regularization. However, the following layers differed between the classifier and the regression models: We did not use a fully connected layer with sigmoid activations for the classifier and instead, a linear transformation was applied to the outputs of the max pooling layer which was followed by a fully connected layer and a single output unit with a sigmoid activation function which outputs a probability of whether the input sequences underlies a GM12878 OCR. We trained the model using SGD to minimize cross-entropy between this output and the binary label of peak/not-a-peak for the training sequences. Again, we selected the best performing model based on cross entropy on the chromosome 4 validation set. We varied the number of convolution layers (2 or 3) following the first layer, the number of filters (filter size 8, stride 1) in these convolution layers (200 or 300), the dropout probability (0, 0.1, 0.4, 0.5, 0.8) for the max pooling layer (pooling size 13, stride = 13) after the final convolution layer, and the learning rate (0.1, or 0.01) for SGD. We used a constant batch size of 64. Each model was trained for 100 passes through the training set. We report the test set performance of the best performing model on the validation set based on mean squared error.

### Reprocessing of Tewhey *et al*. [38] MPRA sequences and training CNN models on MPRA sequences

We downloaded information for all genetic variants and allelic pairs included in the 150bp oligo library with 79,000 sequences from the Tewhey *et al*. [38] MPRA. This included information about the reference allele, alternative allele, the hg19 coordinate for each genetic variant, whether the 150bp oligo was reverse-complemented, and whether the 150bp oligo library included alternative alleles for other genetic variants in the 150bp window. Since the raw sequences were not publicly available, and we needed to construct 1000bp sequences for propagating them through our models, we reconstructed 1000bp sequences by putting the oligo in the center and using hg19 surrounding sequence to make up the rest of the 1000bp of sequence. In places where the original 150bp oligo was reverse-complemented, we reconstructed the reverse complement of the entire 1000bp sequence that we constructed. In addition, where usage of alternative alleles for nearby genetic variants in the 150bp were indicated, we substituted in the alternative alleles for all genetic variants in the 1000 Genomes Phase 1 data that lay within the 1000bp window. To help other researchers create machine learning model training sets using our approach, our code for reconstructing these sequences is made available at https://github.com/pfenninglab/ml-prototypes/tree/public_facing/lcl_regressions. To train models directly on MPRA sequences, we held out all sequences derived from chromosome 3 and chromosome 7 as a validation set and all sequences derived from chromosomes 1,2,4,6,8,10,13,14,15,16,18 and 20 as a test set. We did this to ensure a large enough test set (n = 33,179) to enable robust evaluation of generalization performance. While this left us with a smaller validation set, it was still able to capture n = 7001 sequences to enable robust hyperparameter tuning. We then trained a CNN regression model on the sequences derived from the remaining chromosomes to predict the final expression value [or log2FC (rna/plasmid)] of each sequence (in contrast to the DNase-seq model, which was trained to predict the signal value for open chromatin). We selected the best-performing model based on mean squared error on the validation set. We fixed the number of filters in the first convolution layer to 1000 with a filter size of 8 and a stride of 1. We varied the number of convolution layers (2 or 3) following the first layer, the number of filters (filter size 8, stride 1) in these convolution layers (200 or 300), the dropout probability (0, 0.1, 0.5, or 0.8) for the max pooling layer (pooling size 13, stride = 13) after the final convolution layer, the number of units in the sigmoid layer (100 or 200) that came after the max pooling layer, and the learning rate (0.1, or 0.01) and batch size (30 or 100) for SGD. We trained each model for 100 passes through the training set. We report the test set performance of the best performing model on the validation set based on mean squared error.

### Propagation of reprocessed MPRA sequences through LCL DNase- and MPRA-trained CNN models

For each sequence in the Tewhey 79,000 oligo library, we took the 1000bp sequences constructed earlier and propagated them through the best performing CNN regression model trained on GM12878 DNase-seq, the best performing CNN classifier trained on GM12878 DNase-seq and the best-performing CNN regression model trained on the MPRA data, We then visualized the relationship between the predicted value from each of the three models and the MPRA log2FC value for each sequence in order to evaluate whether the models accurately predict regulatory activity (as measured by the log2FC value in the MPRA). To perform a quantitative evaluation of each model, we computed Pearson’s and Spearman’s correlation between predicted expression and measured expression restricting to those sequences that were included in the MPRA CNN test set.

To test whether models can predict allelic skew, we took each reference/alternative allelic pair in the 79,000 oligo library and compared model outputs for the associated pair of 1000bp sequences. For this analysis, we also included the best-performing LCL task from the Sei multitask model [46], which is an updated version of the DeepSea model [29]. Specifically, we chose the GM12878 DNase-seq task with the best auPRC out of the 6 GM12878 DNase-seq tasks in Sei, which was task ID 6173 and column 13843 in the output.

For the DNase-trained CNN regression and the MPRA-trained CNN regression model, we directly subtracted the model output for the reference allele carrying sequence from the alternative allele carrying sequence to obtain predicted allelic skew values. For the DNase-trained CNN classifier and the Sei model, we first performed an inverse sigmoid (or logit) transformation on the model output, then computed the difference in the transformed scores between the reference allele carrying sequence and the alternative allele carrying sequence. Genetic variants in the 79,000 oligo library were categorized into variants that displayed confident positive allelic skew (|log(skew)|≥0.2, q<0.05) and variants that did not display confident allelic skew (|log(skew)|<0.2, q>0.05). Further, variants that displayed confident allelic skew (or expression modulating variants—“emVars”) were categorized into ones that showed positive allelic skew (i.e. expression for reference-allele-carrying sequence was higher in the assay than the alternative-allele-carrying sequence) and ones that showed negative allelic skew (i.e. expression for reference-allele-carrying sequence was lower in the assay than the alternative-allele-carrying sequence). Then, we used t-tests to test whether our CNN models are able to accurately predict the direction of allelic skew. For emVars with positive skew, we tested whether the mean of predicted allelic skews from eac model was significantly greater than 0 using a one-sample one-sided t-test. For emVars with negative skew, we tested whether the mean of predicted allelic skews from the model was significantly less than 0 using a one-sample one-sided t-test. For neutral variants that did not display a confident allelic skew in the assay, we used a one-sample two-tailed t-test to test whether the mean was significantly different from 0. We plotted the distribution of predicted allelic skews and the p-values from these t-tests for all three models in a single violin plot.

### Transfer learning

Since culturing most non-LCL non-cancer cell lines is challenging, generating large MPRAs from cells related to many diseases is difficult. In practice, only smaller MPRAs may be feasible. Therefore, we evaluated whether we could use a transfer learning approach to train a model with fewer MPRA sequences to achieve better performance at predicting enhancer activity via MPRA than a model trained on DNase-seq data alone. To do this, we trained machine learning models to predict the continuous activity of the sequences that was measured by the Tewhey *et al*. MPRA [38]. We separated the MPRA sequences into training, validation, and test sets, where the training set was derived from chromosomes 17, 11, 19, 12, 5, 22, 9, and 21; the validation set was derived from chromosomes 3 and 7; and the test set was derived from chromosomes 1, 6, 10, 4, 2, 16, 20, 15, 8, 14, 18, and 13. This gave us a training set with 38,512 sequences, a validation set with 7,627 sequences, and a test set with 33,179 sequences. We used 150bp sequences instead of 1,000bp padded sequences because we wanted to evaluate if our transfer learning approach could be used to create a follow-up MPRA with likely enhancer sequences, so we needed to limit our sequence lengths to those on the MPRA. We trained 3 types of models on different sized subsets of the training set. For the first model, we trained on the full training set; for the second model, we used half of the training set and for the third model, we used one quarter of the training set. Since we used the same training/validation/test set as those used for the previous MPRA-trained regression, we started training each CNN model using the architecture from the previous model and then tuned hyper-parameters based on validation set performance, which we evaluated using Pearson and Spearman correlation coefficients between the validation set real activity and predicted activity. We trained CNN models using Keras version 2.2.4 with the theano version 1.0.3 backend, and we evaluated the models using scipy version 1.3.1. We did not downsample the data further because the validation set performance for the model trained on one quarter of the dataset was not especially strong. We used an initial convolution layer with 1000 filters (filter size = 8, stride = 1) with a ReLU activation followed by a variable number of additional convolution layers with ReLU activations and a variable number of filters (filter size = 8, stride = 1) in each layer. We found that 3 additional convolution layers with 150 filters each worked best for the model trained on the full training set. For the model trained on half of the training set, 2 additional convolution layers with 150 filters each worked best. For the model trained on one quarter of the training set, 2 additional convolution layers with 100 filters (filter size = 8, stride = 1) each worked best. In all models, these convolution layers were followed by a max pooling (pooling size = 13, stride = 13) layer. We applied dropout to the output of the max pooling layer with probability 0.8. The outputs of the max pooling layer went into a fully connected layer with a variable number of sigmoid activation units. For the models trained on all and half of the full training set, 100 units worked best for this layer, whereas for the model trained on one quarter of the full training set, 50 units worked best. The output of this layer was fed to a single output unit with no activation. We found that a learning rate of 0.02 and batch size of 100 worked best for all models.

To evaluate if transfer learning could help improve the performance of models trained on one quarter of the training dataset, we tried pre-training CNN regression models on sequences from DNase-seq peaks. To do this, we used IDR reproducible peaks from DNase-seq data from GM12878 from ENCODE (ENCODE Accession IDs: ENCFF057JYL, ENCFF363MQH, ENCFF716JJU, ENCFF855ODQ, ENCFF903PJT) and separated them into training, validation, and test sets using the same chromosomes for each that we used for the MPRA sequences. We added negative sequences to the DNase sequences by using genNullSeqs [32] with settings genomeVersion = ’hg19’, xfold = 2, repeat_match_tol = 0.02, GC_match_tol = 0.02, and length_match_tol = 0 to generate random regions of the genome with approximately the same G/C- and repetitive-element content as our peaks, and we found performance improved with these negative sequences. After hyper-parameter tuning, we found that a CNN model with 2 convolutional layers containing 200 convolutional filters (filter size = 8, stride = 1), followed by a fully connected layer with 100 sigmoid units and dropout 0.8, followed by a linear output, which was trained with learning rate 0.01, momentum 0, and batch size 100 for 100 epochs gave decent DNase-seq validation set performance. We then implemented transfer learning by using this trained model to pretrain a model for predicting MPRA sequences and fine-tuning that model with the same quarter of the MPRA sequences that we used in the corresponding MPRA model. We tuned the learning rate from 0.01 to 0.09 using the MPRA validation set and found that the model with learning rate 0.08 and another 100 epochs worked best.

To evaluate the extent to which transfer learning results depended on which MPRA sequences we sub-sampled, we took the remaining three quarters of the MPRA sequences that did not include the quarter we previously used for fine-tuning and re-did the fine-tuning separately with each of them. We used the same learning rate and number of epochs that achieved the best validation set performance when using the original quarter. We repeated all evaluations for the original quarter on each of the three remaining quarters. We found that results were consistent across quarters of the MPRA sequences used in fine-tuning. To check whether LCL models can identify AD-associated variants and can be used for AD variant effect prediction, we also scored AD-associated variants through the best DNase-seq model and the 4 transfer learning models and cross-referenced the outlier variants detected from these models against the outliers detected by models for other cell types (**Scoring of AD variants for their regulatory effects through CNN models in different cell types).**

### Reprocessing of bulk open chromatin datasets for microglia, CD14+-monocytes, and neurons

Using the ENCODE ATAC-seq pipeline (https://github.com/ENCODE-DCC/atac-seq-pipeline) with default ATAC-seq parameters, we processed all *ex vivo* human microglia ATAC-seq datasets from Gosselin *et al*. [51] downloaded from the dbGaP website, under phs001373.v1.p1 (https://www.ncbi.nlm.nih.gov/projects/gap/cgi-bin/variable.cgi?study_id=phs001373.v1.p1), to generate a set of IDR reproducible human microglia OCRs. Similarly, we processed all human putamen NeuN+ ATAC-seq samples from GSE96949 [56] to generate a set of neuronal OCRs using the ENCODE ATAC-seq pipeline with default parameters except for "atac.multimapping": 0, "atac.cap_num_peak": 300000, "atac.smooth_win": 150, "atac.enable_idr": true, and "atac.idr_thresh": 0.1. We processed CD14+-monocyte DNase-seq samples (ENCODE Accession IDs: ENCFF231SIM, ENCFF000TBP, ENCFF690LNW, ENCFF000TBL) using the Kundaje Lab ATAC-Seq / DNase-Seq Pipeline with default parameters except for -enable_idr to generate a set of monocyte OCRs. For all three cell types, we used the IDR “optimal set” as the final peak set. The optimal set is the larger set out of the IDR reproducible peak set called across replicates and the IDR reproducible peak set called across pooled pseudo-replicates. Overall, we were able to get 181,122 microglia peaks, 126,727 monocyte peaks, and 177,004 neuron peaks to train, validate, and test CNN models.

### Reprocessing of Satpathy *et al*. [49] immune scATAC-seq data

We reprocessed the scATAC-seq dataset in Satpathy *et al*. [49] to look for enrichments in specific immune cell types as well as train CNN regression models on them. Starting with the raw fragment files published in the study, we filtered cells of interest for ArchR [50] peak calling. We converted the fragment files to arrow files with parameters minFrags = 50 to allow for a more relaxed cutoff of minimum number of fragments per cell and get more peaks per cell. These arrow files were then used to create an ArchRProject object to which cluster information was manually added based on grouping of cells into 8 cluster combinations. For each cell type, a subset project was created followed by peak calling for individual cell types. We ran this step to avoid iterative overlap peak removal across cell types which is a default peak calling functionality in ArchR. ArchR first calls peaks within each cell type using MACS2 followed by an iterative merging step to call a superset of peaks across cell types. In the iterative merging step, one peak with the highest signal may be selected and others in the vicinity might be filtered out may lead to loss of important cell type-specific peaks and peak summits. Another important part of ArchR peak calling is creation of pseudo-bulk replicates using addGroupCoverages. The parameters for this method should clearly reflect the number of cells in each cell type and the number of replicates in our dataset, so that ArchR uses all input data. If these are not correct, only a subset of the cells is used to call peaks and we may miss out on some signals. A maxReplicate value of 16 and maxCells value of 4000 was used to allow all data to be used. Peaks were then called using addReproduciblePeakSet with a q-value cutoff of 0.05 and a large enough maxPeaks setting to allow more than the default 150,000 peaks to be called. The natural log of the score generated by MACS2 [80] peak calling was used as the regression label for the called peaks. Peaks on chromosomes 2,20,21,22 were moved from the training to the validation set to get larger sets for evaluating models.

### Stratified LD score regression (S-LDSC) analysis to identify AD-relevant cell types

To identify cell types enriched for AD risk, we conducted stratified LD score regression (S-LDSC) [35–37] analysis on open chromatin and H3K27ac ChIP-seq peaks derived from different cell types. We downloaded DNase-seq peaks for 53 cell types/tissues/cell lines as well as H3K27ac ChIP-seq peaks for 98 cell types/tissues/cell lines in the Epigenome Roadmap database [16]. To the H3K27ac dataset, we added 12 other peak sets corresponding to 3 other cell type populations (microglia, neurons, and oligodendrocyte enriched glial) from the hippocampus and dorsolateral prefrontal cortex ChIP-seq dataset that we have previously analyzed [19]. We then conducted S-LDSC analysis using the approach by the S-LDSC authors first on the DNase-seq dataset testing enrichment for each individual cell type against a background of the other 52 cell types in that dataset. We then extracted the enrichment p-values and corrected them across all 53 tests using Benjamini Hochberg FDR correction (BH FDR). We used a FDR q-value cutoff of 0.05 (q<0.05) to identify enriched cell types. We conducted two such analyses for Jansen *et al*. [9] and Kunkle *et al*. [8] in our analysis. Similarly, we ran S-LDSC analysis on the H3K27ac ChIP-seq dataset applying FDR correction across all 110 peak sets in the H3K27ac dataset. Since we saw enrichments for peripheral immune cell types in these primary DNase-seq and H3K27ac ChIP-seq analysis, we sought to identify which immune cell types are more strongly enriched relative to other cell types by conducting S-LDSC on the Satpathy *et al*. [49] immune scATAC-seq peaks. In this analysis, we ran S-LDSC analysis on peaks for each cell type against a background containing a merged set of peaks for the other 7 cell types. We applied multiple hypothesis correction across all 8 tests using BH FDR correction. To compare peripheral immune cell peaks with brain resident microglial peaks, we conducted a final S-LDSC analysis testing ENCODE CD14+ monocyte peaks against the brain resident microglial peaks from the Gosselin *et al*. [51] study. We extracted and plotted LDSC model coefficients for these peaks sets along with uncorrected p-values for enrichment.

### Training CNN models on bulk open chromatin datasets for microglia, CD14+-monocytes, and neurons

Notably, S-LDSC only looks at colocalization between the genetic association signals in GWAS and the genomic regions of interest. To identify functional variants, it is necessary to show that a variant has an impact on the regulatory activity of an OCR. To achieve this, we trained separate single task CNN regression and classifier models for each dataset, similar to the process described for the LCL DNase-seq data. To obtain negative examples for CNN classifiers, we again used BiasAway [84,85] to generate a set of negative sequences and associated coordinates that matched the G+C-content distribution of our training set sequences. In order to remove potentially false negative sequences before training, we then used bedtools subtract [86] to remove sequences in this negative set that overlapped peaks in each of the individual replicates and in the IDR optimal and conservative sets output by the ENCODE pipeline. We constructed a superset of positive and negative sequences in order to train the model. For all models, we held out sequences on chromosome 4 as a validation set and sequences on chromosomes 8 and 9 as a test set. We used the rest of the sequences for training models. We followed the same model training strategy that we employed for the LCL DNase-seq dataset. To select the best performing architecture and hyperparameters, we evaluated model performance on the chromosome 4 held-out sequences and chose the best-performing model in terms of MSE. We fixed the number of filters in the first convolution layer to 1000 with a filter size of 8 and a stride of 1. We varied the number of convolution layers (2 or 3) following the first layer, the number of filters (filter size 8, stride 1) in these convolution layers (200 or 300), the dropout probability (0, 0.1, 0.5, or 0.8) for the max pooling layer (pooling size 13, stride = 13) after the final convolution layer, the number of units in the sigmoid layer (100 or 200) that came after the max pooling layer, and the learning rate (0.1, or 0.01) and batch size (30 or 100) for SGD. Each model was trained for 100 passes through the training set (or “epochs”). We report the test set performance of the best performing model on the validation set based on mean squared error.

### Training scATAC-seq CNN regression models

We trained 8 models to predict the scATAC-seq signal in the 8 different cell types. Our models took in 500 bp of input sequences and were trained to predict the natural logarithm of the score generated by MACS2 peak calling in ArchR. Our models consisted of an initial convolution layer with 500 units (kernel size = 8, stride = 1) with a rectified linear unit activation (ReLu) followed by a max pooling operation (pooling size = 4, stride = 4). A low dropout regularization value of 0.001 when applied to the first max pooling layer yielded better performance than higher values. This was followed by two more convolution + max pooling blocks. The first of these contained 250 convolution filters (kernel size = 8, stride = 1) with ReLu activations followed by max pooling (pooling size = 4, stride = 4). The next one contained 100 convolution filters (kernel size = 8, stride = 1) with ReLu activations followed by max pooling (pooling size = 4, stride = 4). The output of the pooling operation was flattened and passed into a single output unit which predicts the scATAC-seq peak signal. We applied dropout with probability 0.25 and L2 kernel regularization of 0.01 to the last 2 max pooling layers. We used a cyclic learning rate [87] scheduler for SGD optimization to achieve faster convergence. Training models with an unweighted mean squared error loss function did not lead to satisfactory performance on peaks with higher signal values. Therefore, we used a weighted mean squared error loss with up-weighting for samples with a signal value greater than 4 by a factor of 3. Essentially, this penalized wrong predictions three times as heavily for higher signal instances which our earlier models were failing to predict as opposed to lower signal instances and helped improve model performance. To report final performance, we report Pearson and Spearman’s rank correlation coefficient between model predictions and true scATAC-seq signal values. Additionally, to test whether our models were predicting cell type-specific signals and differentiate between different blood cell types, we selected a cell type (monocyte) and evaluated predictions of the model trained on that cell type on peaks in another cell type (CD4+ T-cells) that did not overlap monocyte peaks. We then compared monocyte model predictions for these peaks with monocyte model predictions for monocyte peaks that did not overlap CD4+ T-cell peaks **(Fig 6E)** using a 2-sided Mann-Whitney test. As another way to test robustness of our scATAC-seq trained models, we compared predictions scATAC-seq model predictions of variant effects for a cell type (monocyte) with predictions of variant effect from models trained on bulk open chromatin datasets from the same cell type (previously trained ENCODE CD14+ bulk DNase-seq model) **(Fig 6F).** We computed Pearson’s and Spearman’s rank correlation between these variant effect predictions to see whether scATAC-seq model predictions agree with bulk open chromatin trained models.

### Constructing a set of LD expanded AD-associated variants

To identify a set of input AD-associated variants that we score for their effects using our CNN models, we curated a set of sentinel AD GWAS variants identified in Jansen *et al*. [9] and Kunkle *et al*. [8] with a significance threshold of 5x10^-8^. We then expanded this list to include variants in linkage disequilibrium (LD) with the sentinel variants. using HaploReg(v4.1) [13], which uses LD information from the 1000 Genomes Project. Using a LD r^2^ threshold of 0.5, we obtained a total of 1,209 variants in the LD expanded set.

### Scoring of AD variants for their regulatory effects in different cell types through CNN models

We scored OCRs overlapping AD-associated variants in the LD-expanded set using 11 CNN regression models. This includes the 3 bulk open chromatin trained models (Gosselin *et al*. microglia [51]), ENCODE CD14+ monocyte, Fullard *et al*. [56] neuron) and the 8 Satpathy *et al*. [49] immune scATAC-seq-trained models. For each variant, we constructed a pair of sequences, one carrying the reference allele and the other carrying the alternate allele. Surrounding sequence was obtained from the hg19 reference genome. To match the 1000bp input sequence length for the 3 bulk-open-chromatin-trained models, we added 499bp to the left of the variant position and 500bp to the right of the variant position. For the scATAC-seq-trained models, we added in 249bp to the left of the variant position and 250bp to the right of the variant position to get input sequences of length 500bp. We then predicted variant effect scores for all 11 models by computing the difference between the model predicted outputs for each of the 1,209 sequence pairs. To interpret and identify significant variant effect scores, we needed a null distribution to compare these scores against. To construct this null distribution, we obtained a full set of variant annotations across the entire genome from our collaborators in the Rudolph Tanzi Lab. For each of the 11 models, we then scored all variant effects in the Tanzi Lab variant set that overlapped peaks in the dataset that was used to train/validate/test the model. We used only OCR-overlapping variants since CNN models are known to have problems generalizing to areas of the input space that are far away from the training set distribution [57]. Our variant effect score distributions were centered at 0 with heavy tails on both sides. The 0-centering is expected since most variants are unlikely to have any effects on regulatory activity. For ease of interpretation, we used properties of the Gaussian distribution to identify outlier variants that have significant effects on regulatory element activity. In our initial analysis, we identified outliers by selecting all OCR-overlapping variants that lie at least 2 standard deviations away from the 0 value. We use this cutoff because around 95% of values lie within 2 standard deviations of the mean of a Gaussian distribution. Roughly, this corresponds to a p-value cutoff of 0.05, which has been widely used in scientific literature ever since it was described in R.A. Fisher’s tables [59,60]. We obtained p-values by taking the z-scores and computing the inverse cumulative distribution function of the gaussian distribution. We then multiple hypothesis corrected across the subset of 1209 AD-associated variants that overlapped an OCR in the given cell type. In secondary analysis, we scored all 1209 AD-associated sequence pairs through all 11 models but focused our interpretation on only outlier variants identified in the initial analysis. We did so to enable comparison of model scores for the previously identified OCR overlapping outlier variants in other cell types where the variant does not overlap an OCR. Due to compute resource constraints, for the transfer learning models and the DNase-seq model in LCLs, we downsampled the set of variants to 2 million and then scored the subset of variants with intersection in the LCL DNase-seq peak set. We confirmed by visual inspection that the shape of the distribution of scores looks similar to the distribution of scores from the other 11 models even with the downsampling and identified outliers using this downsampled distribution.

For all outlier variants, we cross-referenced them against finemapping results obtained from the LocusZoom web service [88,89] which uses a basic Bayesian refinement method. We took the Jansen *et al*. [9] and Kunkle *et al*. [8] GWAS and uploaded results for all variants falling within a 100kb window both 5’ and 3’ of the outlier variant to LocusZoom. We then obtained the posterior inclusion probability (PIP) along with memberships in credible sets from the resulting outputs. Additionally, we cross-referenced outlier variants identified by our models against outlier variants identified in Corces *et al*. 2020 [20]. The finemapping and Corces *et al*. [20] comparisons are included in **Table 2**.

### Model interpretation using DeepSHAP

For specific outlier variants like rs636317 and rs76726049 identified by our ENCODE CD14+ monocyte model, we conducted model interpretation and assigned contribution scores to each input nucleotide using DeepSHAP [64]. Internally, DeepSHAP builds on a connection with DeepLIFT [90] which describes the difference in output from a selected ‘reference’ output in terms of the difference of the input from the some ‘reference’ input. Usually, the reference input represents some neutral or default input that is selected by the user according to the task. We applied DeepSHAP separately on the reference allele carrying sequence and the alternate allele carrying sequence for a given variant to compute contribution scores for every nucleotide in the 1000nt input sequence. We used a reference input of all 0s which corresponds to an input sequence with all unknown nucleotides (“N”). We then plotted DeepSHAP scores for the middle 200nt of the input sequences to enable easy visualization of the central variant location.

## Supporting information

S1 Fig **A.** Cartoon plot showing that common variants implicated in GWAS are likely to have small effects (adapted from Manolio *et al*. 2008 [42]) **B.** Cartoon plot showing the distribution of gene regulatory activities for different genomic regions, most genomic regions display 0 or no regulatory activity and a smaller proportion display high regulatory activity. Common variants are likely to have small effects on regulatory activity rather than completely deplete regulatory activity. CNN regressions which model quantitative regulatory signal may be capable of learning these subtle effects of common variants and might help improve upon binary classifiers which put every active genome region into one bin and learn the difference between inactive and active genomic regions.
(TIFF)

S2 Fig **S-LDSC analysis of immune cell types A.** Bar chart depicting FDR q-values from an S-LDSC analysis on the Kunkle *et al*. [8] AD GWAS and included DNase-seq peaks from 53 cell types/tissues in the Roadmap Epigenomics dataset. Red line indicates q = 0.05, the significance cutoff based on FDR. Tissues are categorized and colored by whether they are derived from brain, blood, or other tissues. **B.** Bar chart depicting FDR q-values from an S-LDSC analysis on the Kunkle *et al*. [8] GWAS and H3K27ac ChIP-seq peaks from 98 cell types/tissues in Roadmap Epigenomics dataset as well as 12 profiles (”Brain New”) from the cell type-specific brain H3K27ac ChIP-seq dataset from Ramamurthy, Welch *et al*. [19]. Tissues are categorized and colored by whether they are derived from an earlier brain dataset from Roadmap Epigenomics [16], the new brain dataset from Ramamurthy, Welch *et al*. [19], blood, or other tissues **C.** Bar plot showing FDR q-values (-log10**-**transformed) from S-LDSC analysis on the Jansen *et al*. [9] AD GWAS and open chromatin peaks for 8 immune cell types from the Satpathy *et al*. [49] scATAC-seq dataset. Red horizontal line indicates q = 0.05. The LDSC FDR is the significance level after FDR correction across the 8 tests. **D.** Individual heatmaps showing coefficients and significance from S-LDSC analysis comparing monocyte/macrophage chromatin accessibility peaks from different sources (ENCODE, Alasoo *et al*. 2018 [22]) to microglia chromatin accessibility datasets from different sources. The top heatmap represents the S-LDSC coefficient of monocyte peaks relative to a microglia**-**only background. The bottom heatmap represents the negated LDSC coefficient of microglia peaks relative to a monocyte**-**only background. The color in bottom heatmap is negated such that yellow represents monocyte enrichment and dark blue represents microglia enrichment. ‘*’ indicates FDR q-value<0.05 and ‘**’ indicates FDR q-value <0.01. We applied Benjamini**-**Hochberg correction across all 48 tests.
(TIFF)

S3 Fig **Conditional S-LDSC analysis for Gosselin *et al*. microglia and ENCODE monocyte datasets A.** Coefficient of enrichment for peaks in each decile of the ENCODE monocyte peak set and each decile of the Gosselin *et al*. [51] microglia peak set computed via S-LDSC compared to a monocyte/microglia merged background for the Kunkle *et al*. [8] GWAS. **B.** Same as **A** but with the Jansen *et al*. [9] GWAS. **C.** same as **A** but using the full ENCODE monocyte peak set as background for the monocyte deciles and using the full Gosselin microglia peak set as background for the microglia deciles. **D.** same as **C** but for the Jansen *et al*. [9] GWAS.
(TIFF)

S4 Fig Replicate concordance baselines for the Fullard *et al*. **NeuN+ dataset, the Gosselin *et al*. microglia dataset, and the ENCODE monocyte DNase-seq dataset A. F.** Scatter plot of true signal values in individual ATAC-seq replicates of the Fullard *et al*. [56] NeuN+ dataset for the held out test set peaks (chromosomes 8 and 9) presented in Fig 5A. **B.** similar to A. but for the Gosselin *et al*. [51] microglia ATAC-seq dataset and the held out peaks presented in Fig 5B. **C.** similar to A. and B. but for the ENCODE monocyte DNase-seq dataset and the held out peaks presented in Fig 5C.
(TIFF)

S5 Fig Performance of scATAC-seq trained CNN regression models for remaining immune cell types Plots show smooth scatter plots of CNN regression model predicted signal vs ArchR defined true peak signal for test set peaks (chromosomes 8 and 9) from peaks called on pseudo bulk samples constructed for A. B cells B. CD8 T cells C. CD4 T cells D. Natural Killer (NK) cells E. Basophils F. Progenitors and G. Dendritic cells.
(TIFF)

S6 Fig Model predictions of variant effect follow Gaussian-like distributions centered at 0 with heavy tails A. Histograms of predicted variant effects for all genomic variants included in the Tanzi variant set from the three CNN regression models trained on the bulk open chromatin datasets (Gosselin *et al*. [51] microglia, ENCODE CD14+ monocyte, Fullard *et al*. [56] Putamen neuron). **B.** Similar to A but for CNN regression models trained on the 8 immune cell type peak sets derived from the Satpathy *et al*. [49] scATAC-seq dataset. **C.** Distribution of scores from the LCL DNase model on the down-sampled set of variants from the Tanzi Lab variant set that overlap OCR peaks in the LCL DNase-seq dataset, distribution of random variables sampled from a Gaussian with the same mean and standard deviation as the LCL DNase scores, and distribution of random variables sampled from a Laplace distribution with the same mean and standard deviation as the LCL DNase scores. Notably, both the Gaussian and the Laplace distributions do not fit perfectly to the heavy-tailed distribution of scores, and hence, any score cutoff we used based on these distributions would be stricter than the corresponding cutoff on the empirical distribution.
(TIFF)

S7 Fig **Scatter plots of model predictions of variant effects from two models for AD associated variants that overlap the merged set of OCRs used to train the two models A.** Scatter plot of variant effect z-scores from the ENCODE CD14+ monocyte model vs the variant effect z-scores from the Gosselin *et al*. [51] microglia model for variants that overlap peaks in either dataset. The shape of each point indicates whether the variant overlaps OCRs in both datasets or whether the variant only overlaps a monocyte OCR or only overlaps a microglia OCR **B-E** same as A but for model pairs and variants that overlap OCRs in corresponding datasets pairs for other cell types.
(TIFF)

S8 Fig DeepSHAP interpretation shows that the monocyte model assigns positive contributions to the reference allele and negative contributions to the alternate allele of rs28834970 and also focuses on adjacent nucleotides part of a CEBP motif From top to bottom. DeepSHAP contribution scores for the middle 200 bp of for the 1000 bp reference allele carrying sequence. One hot encoding of the middle 200bp of the reference allele carrying sequence. DeepSHAP contribution scores for the middle 200 bp of for the 1000 bp alternate allele carrying sequence. One hot encoding of the middle 200bp of the alternate allele carrying sequence.
(TIFF)

S9 Fig DeepSHAP contribution scores show that the monocyte model focuses on the reference allele as well as adjacent nucleotides on the reference allele carrying sequence for rs7626049. From top to bottom. DeepSHAP contribution scores for the middle 200 bp of for the 1000 bp reference allele carrying sequence. One hot encoding of the middle 200bp of the reference allele carrying sequence. DeepSHAP contribution scores for the middle 200 bp of for the 1000 bp alternate allele carrying sequence. One hot encoding of the middle 200bp of the alternate allele carrying sequence.
(TIFF)

S10 Fig Transfer learning on DNase and MPRA improves prediction of MPRA regulatory activity A. Overview of our transfer learning approach. An initial CNN regression model is pretrained on DNase-seq data and fine-tuned on MPRA sequences. **B.** Bar chart of Pearson’s and Spearman’s correlation values between true and predicted regulatory activity on sequences in the MPRA test set for MPRA-only models trained on different sized subsets (full, half, and quarter) of the MPRA training dataset. **C.** Bar chart of Pearson’s and Spearman’s correlation values between true and predicted regulatory activity on sequences in the MPRA test set for 4 transfer learning model fine-tuned on different quarters (25%) of the MPRA training dataset, a non-fine-tuned model trained only on DNase-seq, and a model trained on only the quarter subset of the MPRA training datasets.
(TIFF)

S11 Fig Variant effect prediction performance of transfer learning models A. Violin plot of variant effect predictions for variants in the Tewhey *et al*. [38] MPRA from all 4 transfer learning models fine-tuned on different subsets of the MPRA training set as well as a DNase-only regression model trained with the same context length. Variants are categorized into skew positive emVars, skew negative emVars, and neutral variants as in **Fig 3.** The p-values displayed on the plot were computed using a one-sided Wilcoxon rank-sum test comparing the distribution of variant effect scores of each transfer learning model to the distribution of variant effects scores of the DNase-only model. **B.** Distribution of variant effect scores from each of the 4 transfer learning models trained on a quarter of the MPRA training set as well as the DNase-only regression model used in pre-training. Model predictions of variant effects again follow a Gaussian-like distribution with a heavy tail.
(TIFF)

S12 Fig DeepSHAP contribution scores show that the monocyte model assigns positive contribution scores to the reference allele and surrounding nucleotides on the reference allele carrying sequence and negative contribution scores to the alternate allele as well as adjacent nucleotides on the alternate allele carrying sequence for rs636317 From top to bottom. DeepSHAP contribution scores for the middle 200 bp of for the 1000 bp reference allele carrying sequence. One hot encoding of the middle 200bp of the reference allele carrying sequence. DeepSHAP contribution scores for the middle 200 bp of for the 1000 bp alternate allele carrying sequence. One hot encoding of the middle 200bp of the alternate allele carrying sequence.
(TIFF)
