# Supplementary figures and images for: Regression convolutional neural network models implicate peripheral immune regulatory variants in the predisposition to Alzheimer’s disease
Source: PLoS Comput Biol. 2024 Aug 26;20(8):e1012356. doi: 10.1371/journal.pcbi.1012356 (PMC11389932; doi:10.1371/journal.pcbi.1012356)

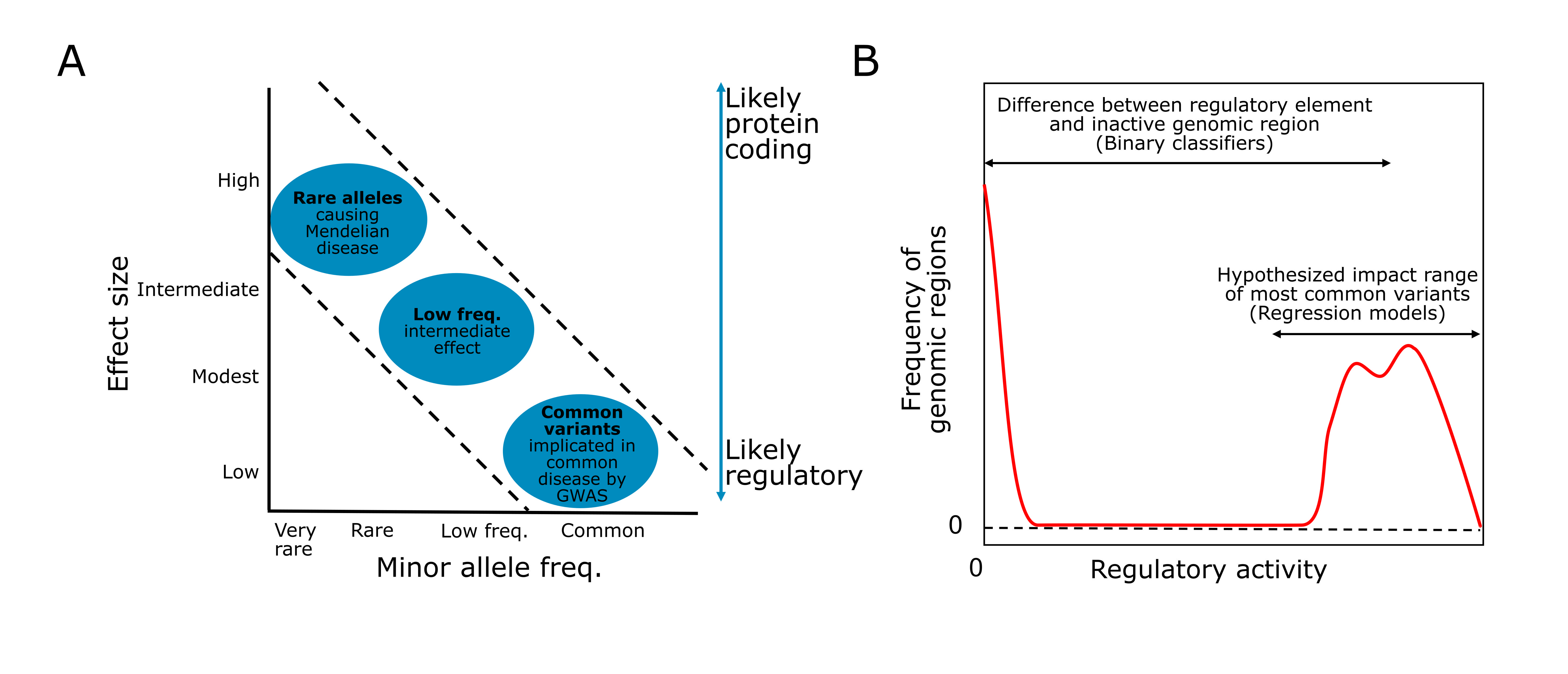

Supplement: S1 Fig — A. Cartoon plot showing that common variants implicated in GWAS are likely to have small effects (adapted from Manolio et al. 2008 [42]) B. Cartoon plot showing the distribution of gene regulatory activities for different genomic regions, most genomic regions display 0 or no regulatory activity and a smaller proportion display high regulatory activity. Common variants are likely to have small effects on regulatory activity rather than completely deplete regulatory activity. CNN regressions which model quantitative regulatory signal may be capable of learning these subtle effects of common variants and might help improve upon binary classifiers which put every active genome region into one bin and learn the difference between inactive and active genomic regions. (TIFF) [file pcbi.1012356.s001.tiff]

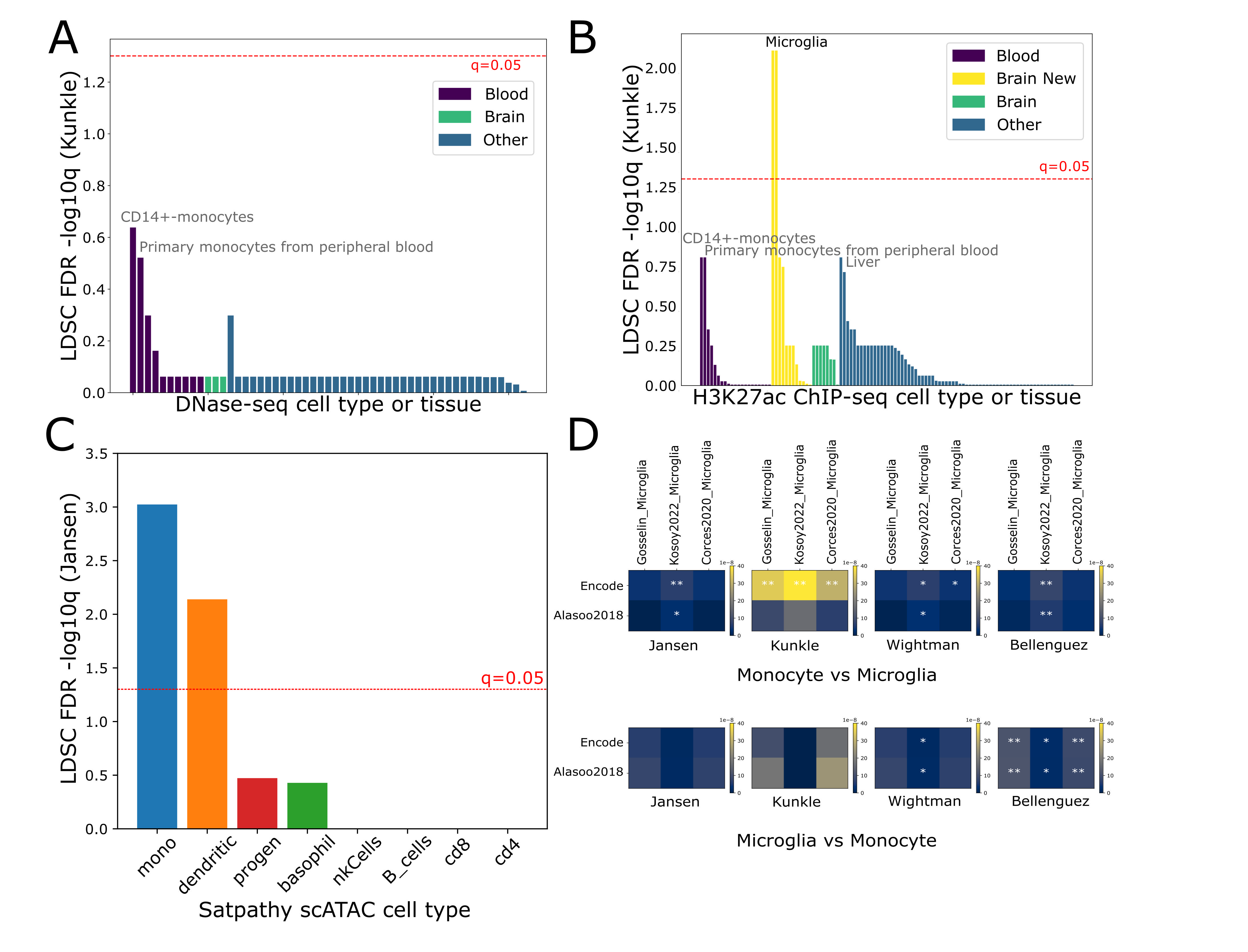

Supplement: S2 Fig — S-LDSC analysis of immune cell types A. Bar chart depicting FDR q-values from an S-LDSC analysis on the Kunkle et al. [8] AD GWAS and included DNase-seq peaks from 53 cell types/tissues in the Roadmap Epigenomics dataset. Red line indicates q = 0.05, the significance cutoff based on FDR. Tissues are categorized and colored by whether they are derived from brain, blood, or other tissues. B. Bar chart depicting FDR q-values from an S-LDSC analysis on the Kunkle et al. [8] GWAS and H3K27ac ChIP-seq peaks from 98 cell types/tissues in Roadmap Epigenomics dataset as well as 12 profiles (”Brain New”) from the cell type-specific brain H3K27ac ChIP-seq dataset from Ramamurthy, Welch et al. [19]. Tissues are categorized and colored by whether they are derived from an earlier brain dataset from Roadmap Epigenomics [16], the new brain dataset from Ramamurthy, Welch et al. [19], blood, or other tissues C. Bar plot showing FDR q-values (-log10-transformed) from S-LDSC analysis on the Jansen et al. [9] AD GWAS and open chromatin peaks for 8 immune cell types from the Satpathy et al. [49] scATAC-seq dataset. Red horizontal line indicates q = 0.05. The LDSC FDR is the significance level after FDR correction across the 8 tests. D. Individual heatmaps showing coefficients and significance from S-LDSC analysis comparing monocyte/macrophage chromatin accessibility peaks from different sources (ENCODE, Alasoo et al. 2018 [22]) to microglia chromatin accessibility datasets from different sources. The top heatmap represents the S-LDSC coefficient of monocyte peaks relative to a microglia-only background. The bottom heatmap represents the negated LDSC coefficient of microglia peaks relative to a monocyte-only background. The color in bottom heatmap is negated such that yellow represents monocyte enrichment and dark blue represents microglia enrichment. ‘*’ indicates FDR q-value<0.05 and ‘**’ indicates FDR q-value <0.01. We applied Benjamini-Hochberg correction across all 48 tests. (TIFF) [file pcbi.1012356.s002.tiff]

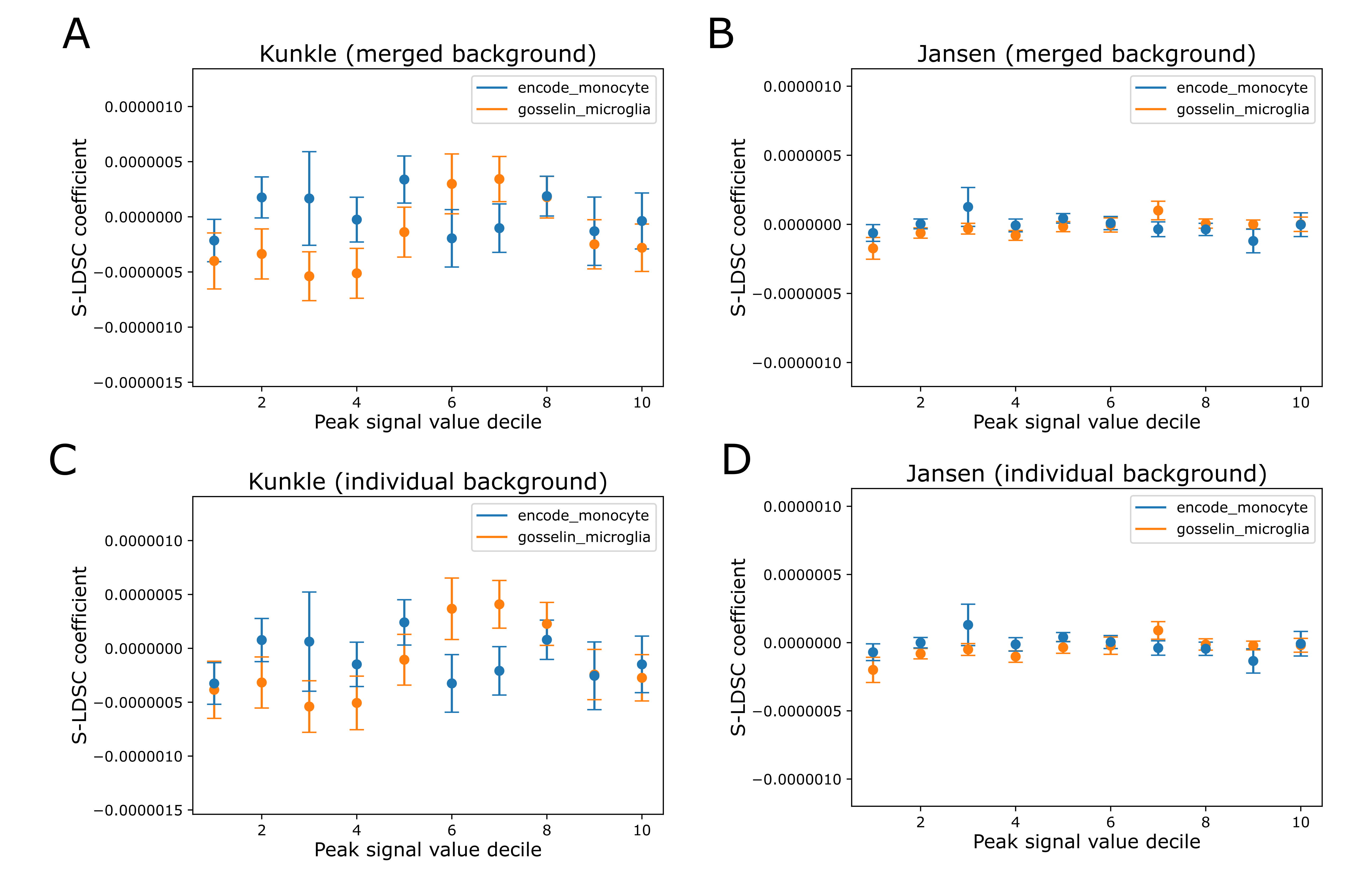

Supplement: S3 Fig — Conditional S-LDSC analysis for Gosselin et al. microglia and ENCODE monocyte datasets A. Coefficient of enrichment for peaks in each decile of the ENCODE monocyte peak set and each decile of the Gosselin et al. [51] microglia peak set computed via S-LDSC compared to a monocyte/microglia merged background for the Kunkle et al. [8] GWAS. B. Same as A but with the Jansen et al. [9] GWAS. C. same as A but using the full ENCODE monocyte peak set as background for the monocyte deciles and using the full Gosselin microglia peak set as background for the microglia deciles. D. same as C but for the Jansen et al. [9] GWAS. (TIFF) [file pcbi.1012356.s003.tiff]

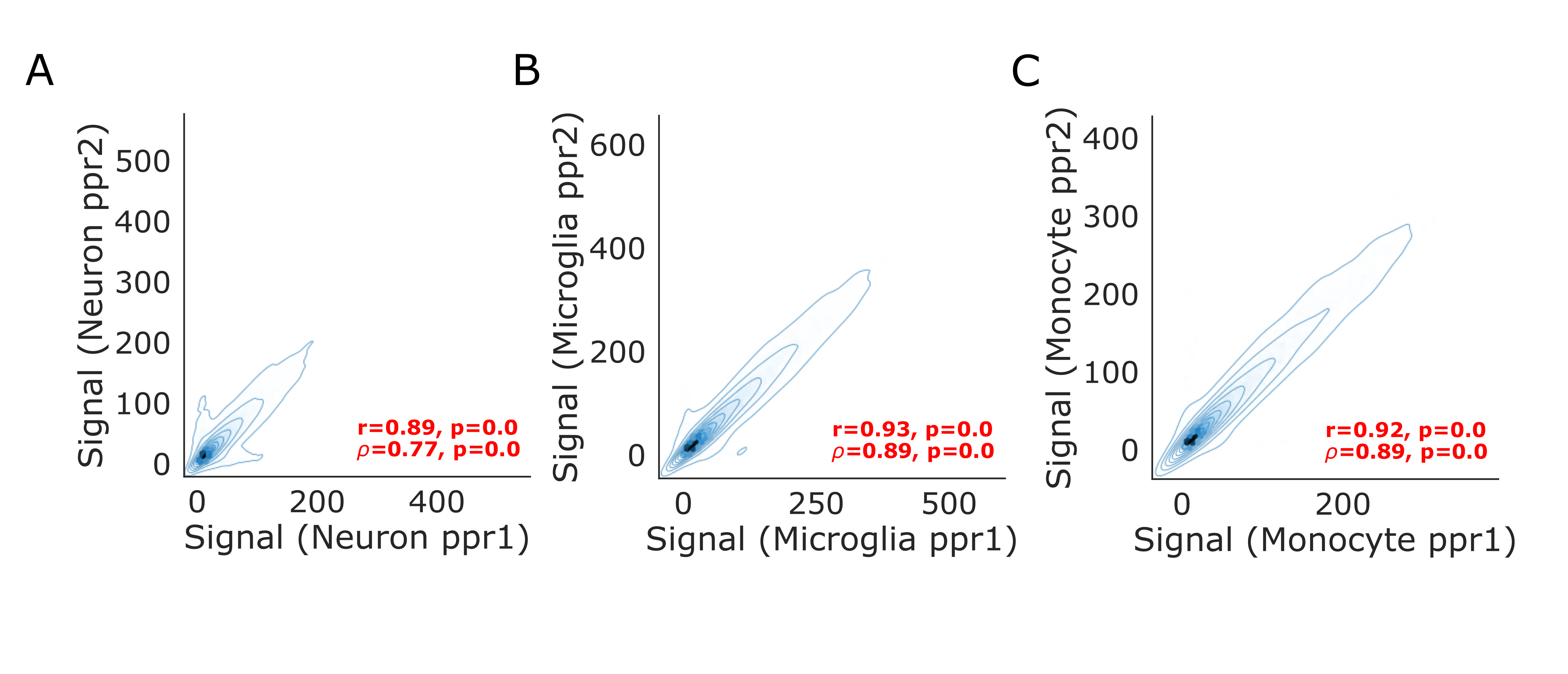

Supplement: S4 Fig — NeuN+ dataset, the Gosselin et al. microglia dataset, and the ENCODE monocyte DNase-seq dataset A. F. Scatter plot of true signal values in individual ATAC-seq replicates of the Fullard et al. [56] NeuN+ dataset for the held out test set peaks (chromosomes 8 and 9) presented in Fig 5A. B. similar to A. but for the Gosselin et al. [51] microglia ATAC-seq dataset and the held out peaks presented in Fig 5B. C. similar to A. and B. but for the ENCODE monocyte DNase-seq dataset and the held out peaks presented in Fig 5C. (TIFF) [file pcbi.1012356.s004.tiff]

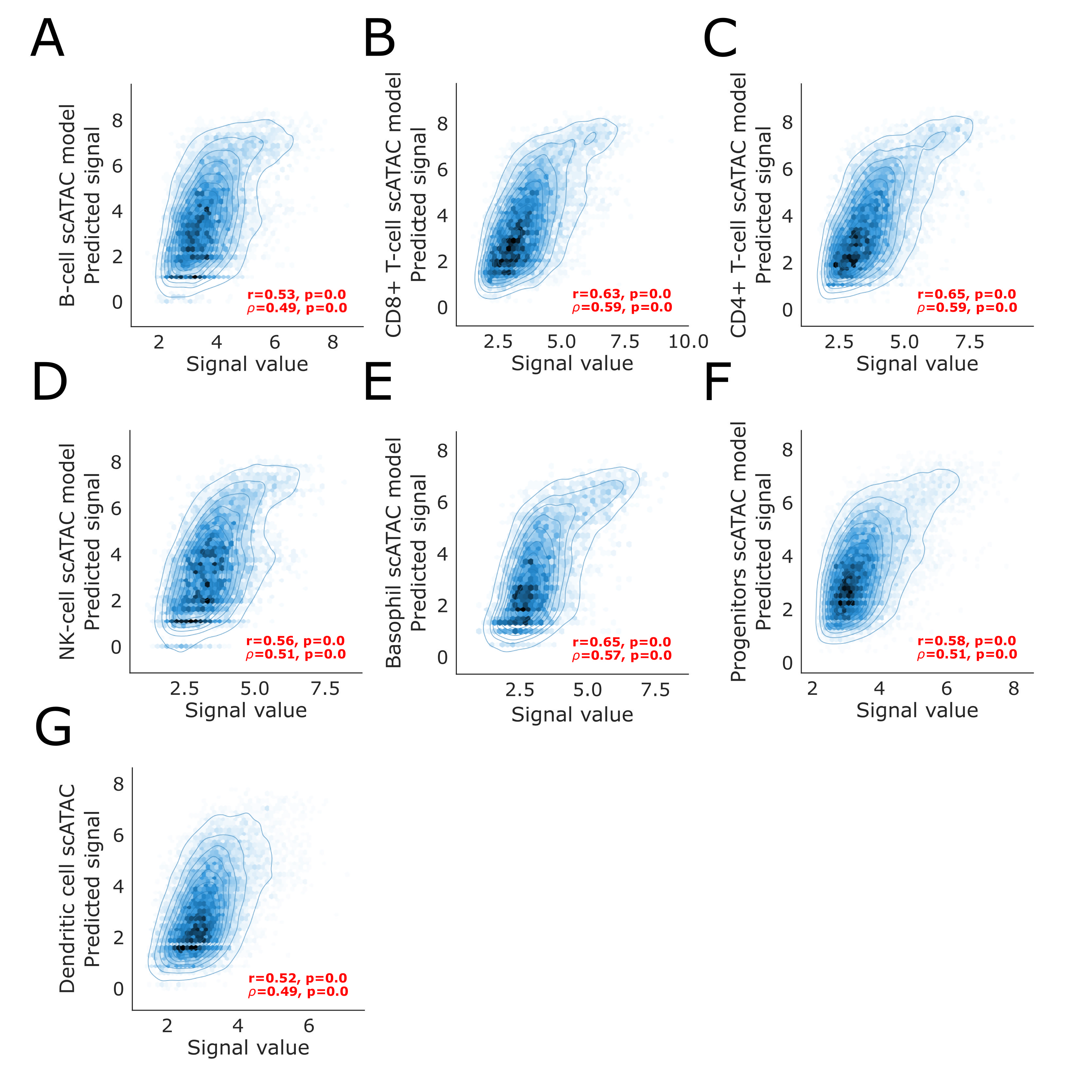

Supplement: S5 Fig — Performance of scATAC-seq trained CNN regression models for remaining immune cell types Plots show smooth scatter plots of CNN regression model predicted signal vs ArchR defined true peak signal for test set peaks (chromosomes 8 and 9) from peaks called on pseudo bulk samples constructed for A. B cells B. CD8 T cells C. CD4 T cells D. Natural Killer (NK) cells E. Basophils F. Progenitors and G. Dendritic cells. (TIFF) [file pcbi.1012356.s005.tiff]

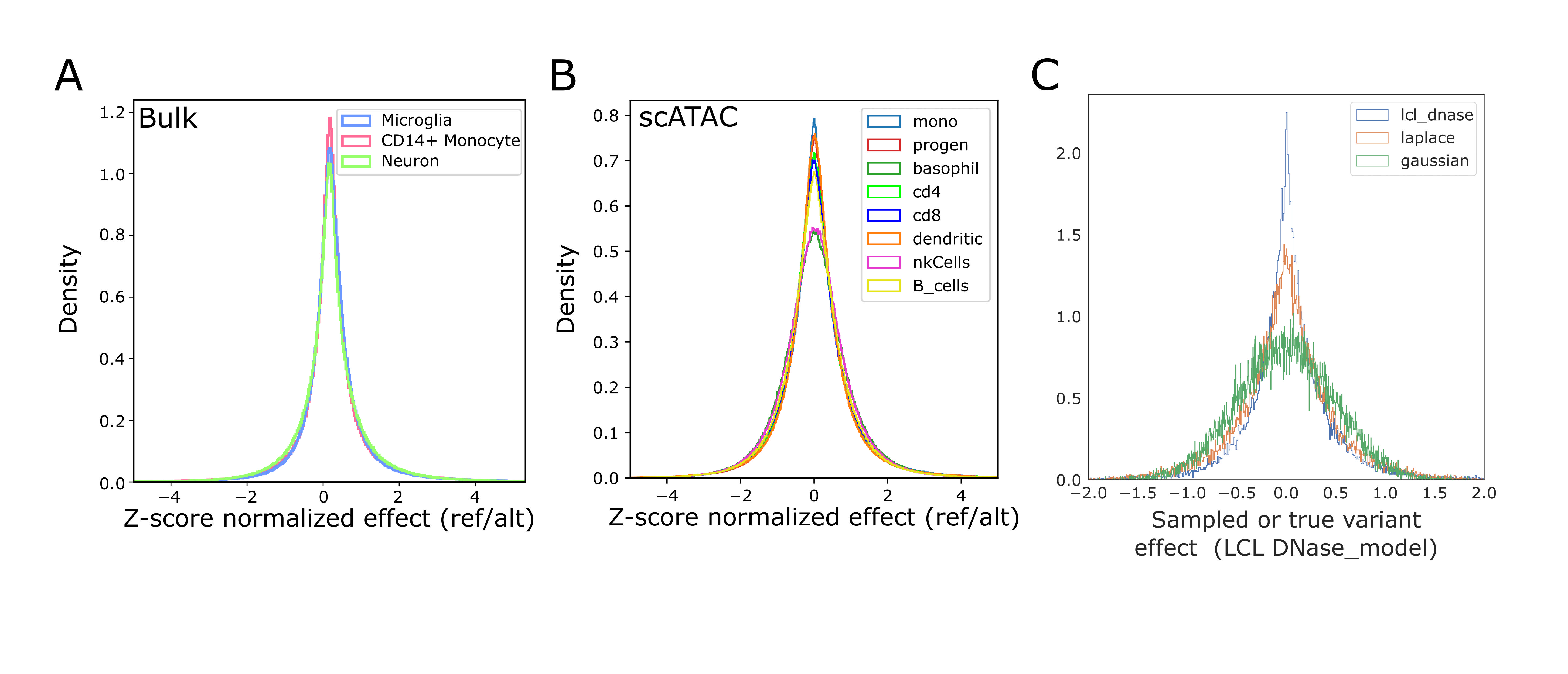

Supplement: S6 Fig — Histograms of predicted variant effects for all genomic variants included in the Tanzi variant set from the three CNN regression models trained on the bulk open chromatin datasets (Gosselin et al. [51] microglia, ENCODE CD14+ monocyte, Fullard et al. [56] Putamen neuron). B. Similar to A but for CNN regression models trained on the 8 immune cell type peak sets derived from the Satpathy et al. [49] scATAC-seq dataset. C. Distribution of scores from the LCL DNase model on the down-sampled set of variants from the Tanzi Lab variant set that overlap OCR peaks in the LCL DNase-seq dataset, distribution of random variables sampled from a Gaussian with the same mean and standard deviation as the LCL DNase scores, and distribution of random variables sampled from a Laplace distribution with the same mean and standard deviation as the LCL DNase scores. Notably, both the Gaussian and the Laplace distributions do not fit perfectly to the heavy-tailed distribution of scores, and hence, any score cutoff we used based on these distributions would be stricter than the corresponding cutoff on the empirical distribution. (TIFF) [file pcbi.1012356.s006.tiff]

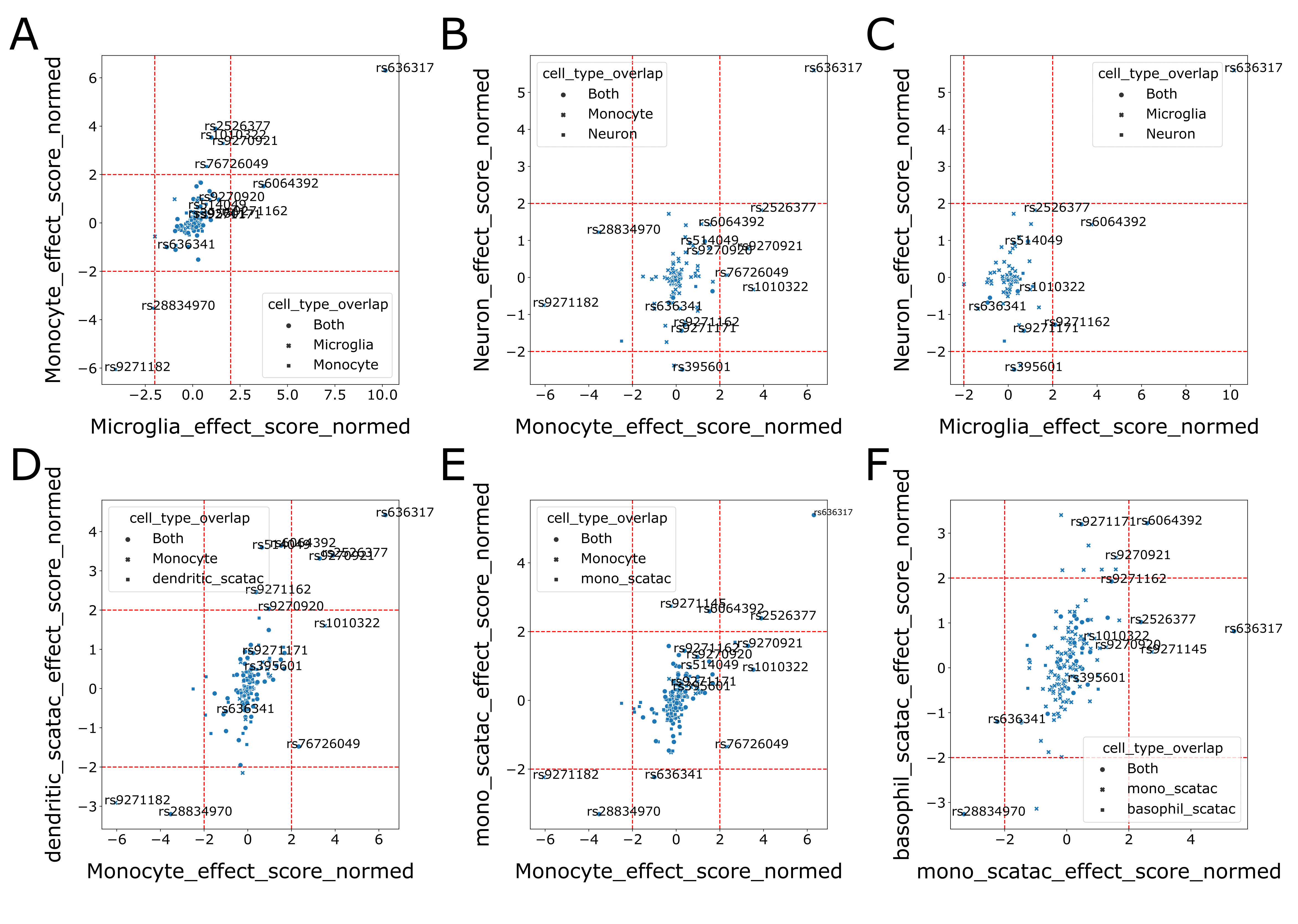

Supplement: S7 Fig — Scatter plots of model predictions of variant effects from two models for AD associated variants that overlap the merged set of OCRs used to train the two models A. Scatter plot of variant effect z-scores from the ENCODE CD14+ monocyte model vs the variant effect z-scores from the Gosselin et al. [51] microglia model for variants that overlap peaks in either dataset. The shape of each point indicates whether the variant overlaps OCRs in both datasets or whether the variant only overlaps a monocyte OCR or only overlaps a microglia OCR B-E same as A but for model pairs and variants that overlap OCRs in corresponding datasets pairs for other cell types. (TIFF) [file pcbi.1012356.s007.tiff]

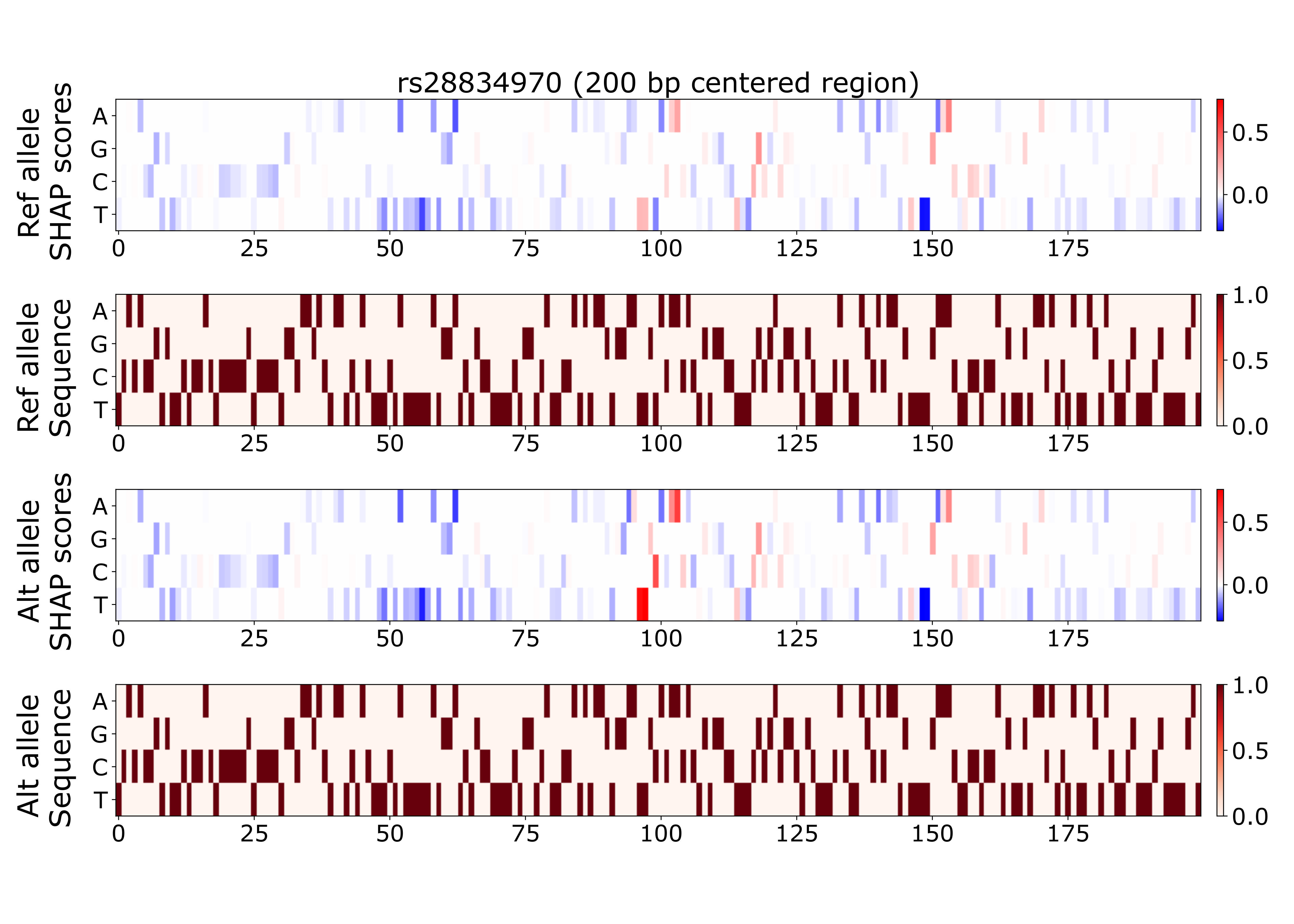

Supplement: S8 Fig — DeepSHAP contribution scores for the middle 200 bp of for the 1000 bp reference allele carrying sequence. One hot encoding of the middle 200bp of the reference allele carrying sequence. DeepSHAP contribution scores for the middle 200 bp of for the 1000 bp alternate allele carrying sequence. One hot encoding of the middle 200bp of the alternate allele carrying sequence. (TIFF) [file pcbi.1012356.s008.tiff]

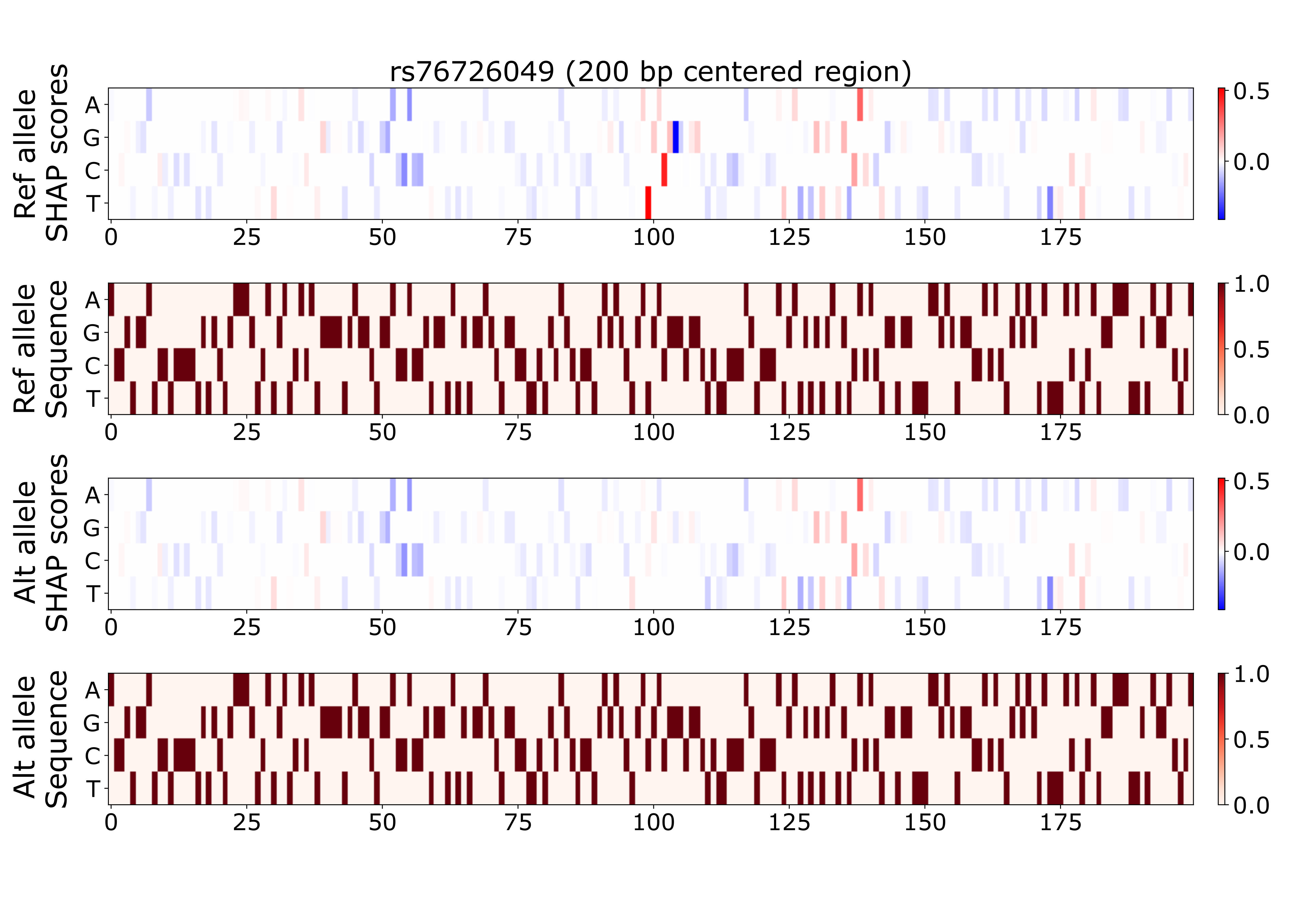

Supplement: S9 Fig — From top to bottom. DeepSHAP contribution scores for the middle 200 bp of for the 1000 bp reference allele carrying sequence. One hot encoding of the middle 200bp of the reference allele carrying sequence. DeepSHAP contribution scores for the middle 200 bp of for the 1000 bp alternate allele carrying sequence. One hot encoding of the middle 200bp of the alternate allele carrying sequence. (TIFF) [file pcbi.1012356.s009.tiff]

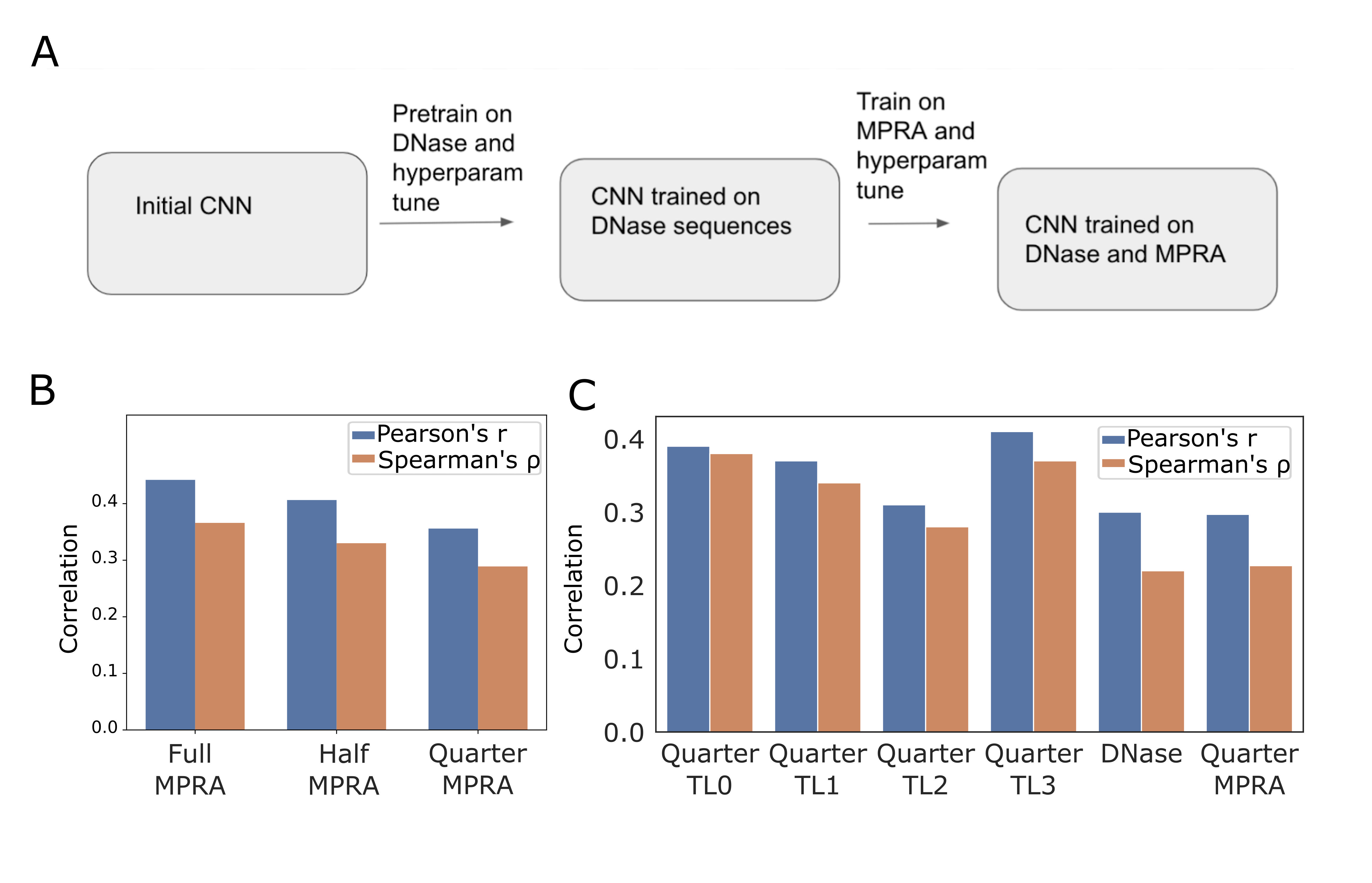

Supplement: S10 Fig — Overview of our transfer learning approach. An initial CNN regression model is pretrained on DNase-seq data and fine-tuned on MPRA sequences. B. Bar chart of Pearson’s and Spearman’s correlation values between true and predicted regulatory activity on sequences in the MPRA test set for MPRA-only models trained on different sized subsets (full, half, and quarter) of the MPRA training dataset. C. Bar chart of Pearson’s and Spearman’s correlation values between true and predicted regulatory activity on sequences in the MPRA test set for 4 transfer learning model fine-tuned on different quarters (25%) of the MPRA training dataset, a non-fine-tuned model trained only on DNase-seq, and a model trained on only the quarter subset of the MPRA training datasets. (TIFF) [file pcbi.1012356.s010.tiff]

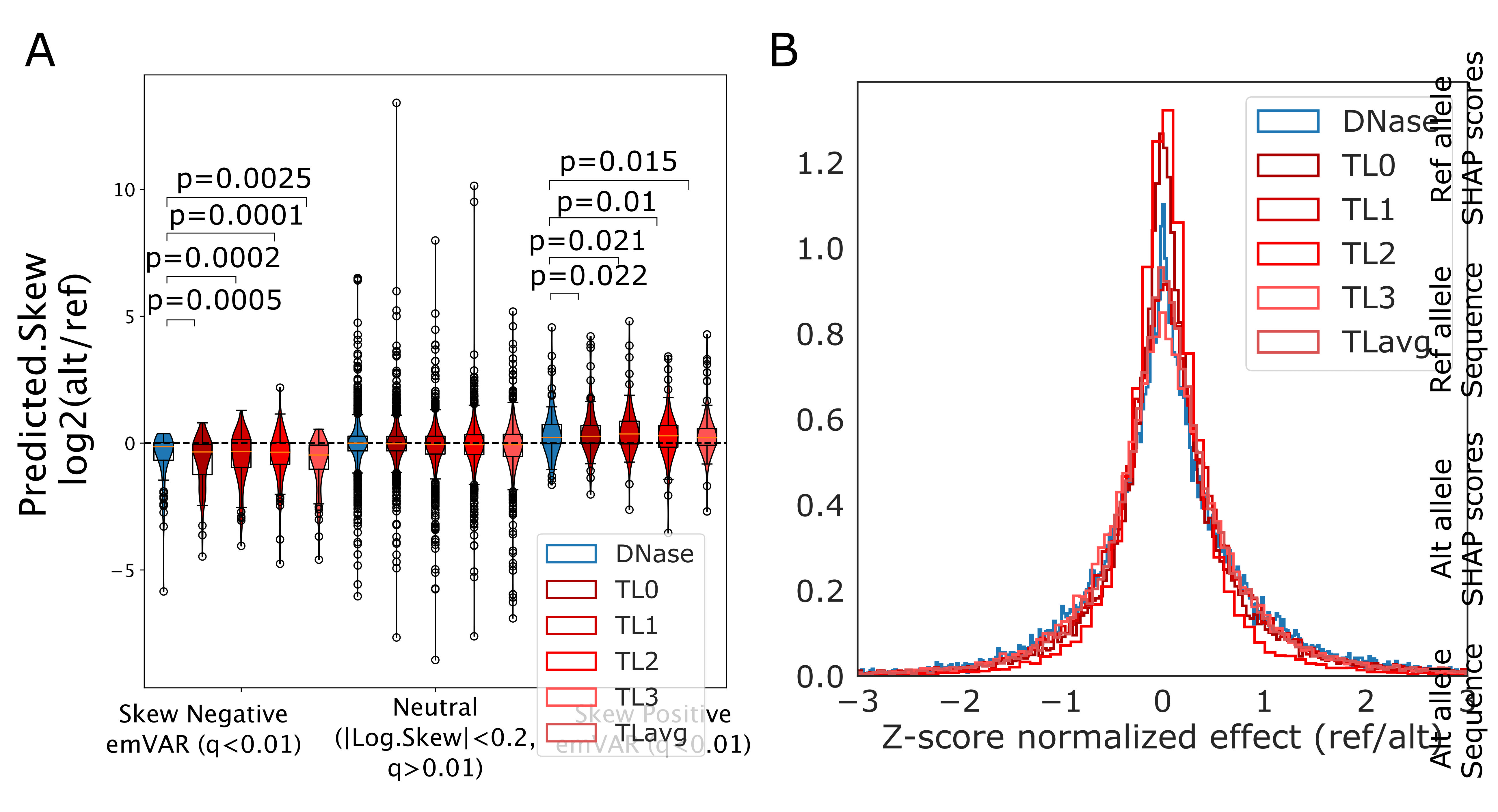

Supplement: S11 Fig — Violin plot of variant effect predictions for variants in the Tewhey et al. [38] MPRA from all 4 transfer learning models fine-tuned on different subsets of the MPRA training set as well as a DNase-only regression model trained with the same context length. Variants are categorized into skew positive emVars, skew negative emVars, and neutral variants as in Fig 3. The p-values displayed on the plot were computed using a one-sided Wilcoxon rank-sum test comparing the distribution of variant effect scores of each transfer learning model to the distribution of variant effects scores of the DNase-only model. B. Distribution of variant effect scores from each of the 4 transfer learning models trained on a quarter of the MPRA training set as well as the DNase-only regression model used in pre-training. Model predictions of variant effects again follow a Gaussian-like distribution with a heavy tail. (TIFF) [file pcbi.1012356.s011.tiff]

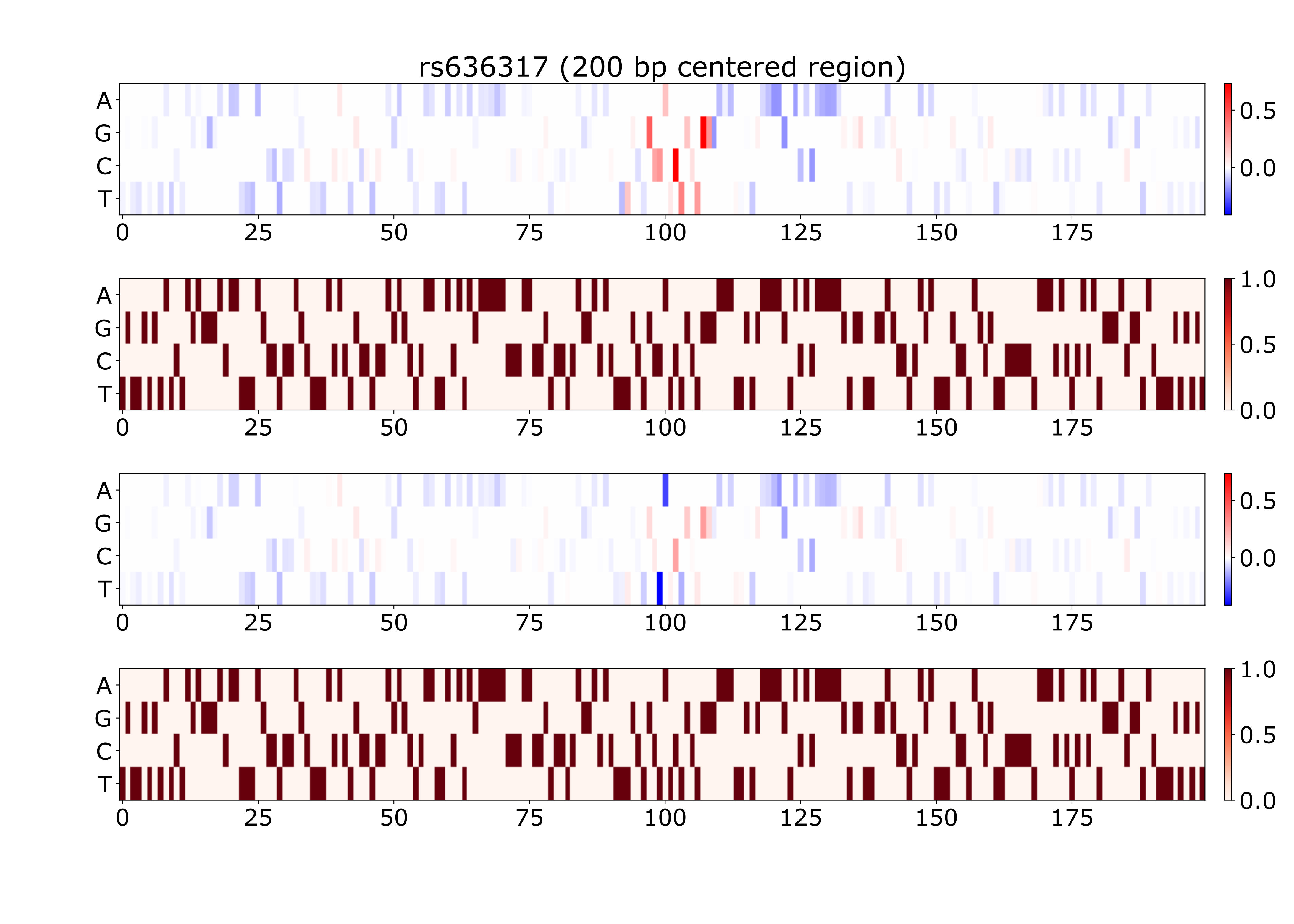

Supplement: S12 Fig — DeepSHAP contribution scores for the middle 200 bp of for the 1000 bp reference allele carrying sequence. One hot encoding of the middle 200bp of the reference allele carrying sequence. DeepSHAP contribution scores for the middle 200 bp of for the 1000 bp alternate allele carrying sequence. One hot encoding of the middle 200bp of the alternate allele carrying sequence. (TIFF) [file pcbi.1012356.s012.tiff]
